# Targeting pain where it Cou*Nts:* the role of neurotensin signaling in pain and analgesia

**DOI:** 10.3389/fphar.2026.1907900

**Published:** 2026-10-14

**Authors:** Alex D. Chapman, Katherine L. Black, Gina M. Leinninger, Geoffroy Laumet

**Affiliations:** 1 Neuroscience Program, Michigan State University, East Lansing, MI, United States; 2 Department of Physiology, Michigan State University, East Lansing, MI, United States

**Keywords:** analgesia, GPCR, neuropharmacology, neurotensin, nociception, pain

## Abstract

Chronic pain affects more than one-fifth of adults in the United States and represents a major public health burden due to its impact on quality of life, productivity, and healthcare costs. Current pharmacological treatments remain limited in efficacy and are often associated with significant adverse effects, highlighting the need for safer and more effective therapeutic strategies. Neurotensin (Nts), a neuropeptide widely expressed in the central and peripheral nervous systems, has emerged as an important modulator of nociceptive processing. Nts signals through the G protein-coupled receptors neurotensin receptor 1 (NTSR1) and neurotensin receptor 2 (NTSR2), which are expressed in key neuroanatomical components of pain pathways. In this review, we summarize current knowledge of the neuronal and molecular mechanisms underlying pain processing and discuss how Nts signaling modulates nociceptive transmission at peripheral, spinal, and supraspinal levels. In addition to regulating nociceptor excitability and spinal excitatory neurotransmission, Nts critically regulates neural circuits involved in descending pain modulation, including the periaqueductal gray and rostral ventromedial medulla, and contributes to pain processing within limbic and hypothalamic networks that integrate sensory and affective components of pain. Finally, we discuss recent advances in pharmacological modulation of Nts receptors, including biased allosteric modulators and receptor-selective agonists that show promising analgesic efficacy in preclinical models. Together, these findings position the neurotensin system as a promising target for the development of novel non-opioid therapies for pain.

## Introduction to pain

Pain, which the International Association for the Study of Pain defines as “An unpleasant sensory and emotional experience associated with, or resembling that associated with, actual or potential tissue damage”, is an important physiological response that helps to alert us of illness and prevent the worsening of existing injuries (Raja et al., 2020). Acute pain is an essential protective mechanism that enables rapid responses to potentially harmful stimuli, including extreme temperatures, noxious mechanical inputs, or inflammation associated with infection (Ji et al., 2016). Beyond its immediate sensory component, pain also encompasses emotional and cognitive dimensions, influencing motivation, behavior, and memory formation related to aversive experiences (Bushnell et al., 2013; Ng et al., 2018; Qiu et al., 2026). These integrated responses are critical for promoting survival and facilitating appropriate protective actions (Walters et al., 2023). However, when acute pain fails to resolve and transitions into chronic pain it becomes maladaptive and often debilitating (Bushnell et al., 2013). Chronic pain, defined as pain persisting for more than 3 months, affects over one-fifth of adults in the United States (Rikard et al., 2023; Yong et al., 2022). This condition imposes a substantial burden, impairing mood, physical function, and daily activities, while contributing to an estimated annual economic cost exceeding $720 billion due to healthcare expenses and lost productivity (Guy et al., 2025). Despite its high prevalence, current treatments for chronic pain remain limited. Many available therapies provide insufficient pain relief, lead to tolerance, or are associated with significant adverse effects, including addiction and organ toxicities (Finnerup et al., 2015; Pulskamp et al., 2024). These limitations underscore a critical unmet need to better understand the mechanisms underlying chronic pain and to identify safer, more effective therapeutic targets. This review will discuss the neural systems that transduce and modulate pain, how the neuropeptide neurotensin (Nts) signals via its G protein-coupled receptor (GPCR)s to modulate pain processing, and the therapeutic potential of the Nts system for treating pain.

### Neural pathways of pain

Pain begins with the activation of pain-sensing primary afferent neurons, termed nociceptors, whose cell bodies reside in the dorsal root ganglia (DRG) and trigeminal ganglia (TG). Peripheral afferents from the DRG and TG innervate tissues throughout the body, where they detect noxious stimuli, including extreme temperatures, chemical irritants, mechanical trauma, and inflammatory mediators (Bridges et al., 2001; Meacham et al., 2017; Novakovic et al., 1998). The DRG are located bilaterally along the vertebral column adjacent to the spinal cord, with each ganglion ensheathed by the spinal dura, and their peripheral fibers innervate nearly all organs below the neck. In contrast, the TG are located within Meckel’s cave of the temporal bone and innervate the head, face, and meninges (Basbaum et al., 2009). Following tissue injury or noxious stimulation, endogenous mediators are released and bind to receptors expressed by nociceptors, including G protein-coupled receptors (GPCRs), initiating action potentials that are transmitted to neurons in the superficial laminae of the spinal dorsal horn (Li et al., 2017). These primary afferent neurons, which include unmyelinated C fibers and thinly myelinated Aδ fibers, respond to noxious stimuli by generating action potentials that are transmitted to neurons in the superficial laminae of the spinal dorsal horn (Basbaum et al., 2009). The second order neurons cross the spinal cord via the ascending tracts to the contralateral part of the thalamus (Basbaum et al., 2009). Centrally, brain regions such as the thalamus and somatosensory cortex relay nociceptive inputs from the periphery (Bausbaum et al., 2009) (Figure 1). Although the spinothalamic tract is the most characterized, other ascending tracts exist such as the spinoparabrachial and parabrachial-amygdala pathways, connecting the spinal cord and emotional centers of the brainstem (Barik et al., 2018), the spinoreticular tract carries arousal and alertness responses associated with pain, and spinohypothalamic tract is involved in regulating autonomic and endocrine response to pain (Han et al., 2015; Willis and Westlund, 1997). This ascending input is balanced by the presence of inhibitory interneurons in the spinal dorsal horn (Ossipov et al., 2010). While peripheral GPCRs are easier to pharmacologically access than central targets, limiting analgesic strategies to peripheral nociceptors carries the risk of primarily suppressing reflexive nociceptive signaling without adequately addressing the affective and cognitive components of chronic pain (Schmidt et al., 2025; Uniyal et al., 2023). Pain causes physical sensations, emotional experiences, impacts our motivation to perform tasks, and creates intense memories related to what caused pain (Bushnell et al., 2013; Ng et al., 2018 Qiu et al., 2026). Thus, regions of the brain often associated with emotion, cognition, and memory also contribute to the conscious perception of pain, such as the amygdala, bed nucleus of the stria terminalis (BNST), ventral tegmental area (VTA), lateral hypothalamic area (LHA), periaqueductal gray (PAG), and the rostral ventromedial medulla (RVM) (Aimone et al., 1988; Barbaro, 1988; Dafny et al., 1996; Lopez et al., 1991; Mayer et al., 1971; Reynolds, 1969; Zhang et al., 2025).

**FIGURE 1:**
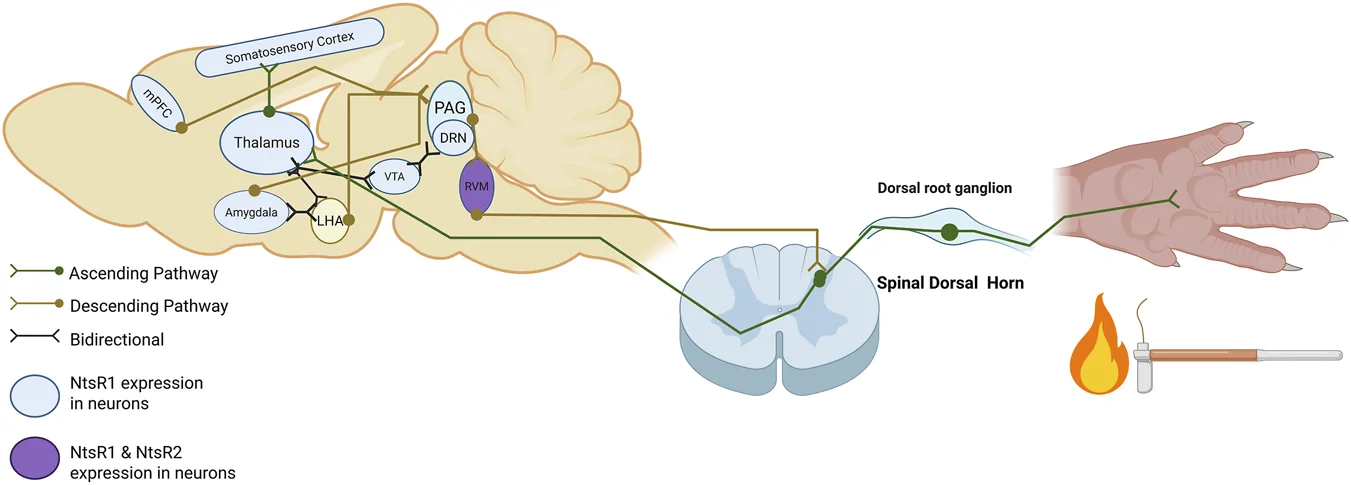
Neuroanatomical pathways and neurotensin receptor expression associated with pain transmission and modulation. Nociceptive information originating in peripheral tissues is transmitted by primary sensory neurons whose cell bodies reside in the dorsal root ganglion (DRG) and project to the spinal dorsal horn (SDH). Ascending pain pathways (green) relay sensory information from the SDH to the brain, including the thalamus and somatosensory cortex, where pain is perceived and discriminated. Descending modulatory pathways (gold) originate from higher-order brain regions, including the medial prefrontal cortex (mPFC), amygdala, lateral hypothalamic area (LHA), periaqueductal gray (PAG), dorsal raphe nucleus (DRN), and rostral ventromedial medulla (RVM), and regulate nociceptive transmission at the level of the spinal cord. Bidirectional connections (black) indicate reciprocal communication between pain-processing regions. Brain regions containing neurotensin receptor 1 (NtsR1)- expressing neurons are highlighted in blue, while regions containing neurons expressing both NtsR1- and NtsR2 are shown in purple. NtsR2 is also expressed by astrocytes throughout the central nervous system.

### Preclinical models to study pain

The study of pain mechanisms and the identification of novel therapeutics rely heavily on preclinical behavioral assays. A variety of tests have been developed to evaluate pain-related behaviors in rodent models (Gregory et al., 2013; Sadler et al., 2022). The most commonly used approaches are stimulus-evoked assays, which measure paw or tail withdrawal responses to thermal or mechanical stimuli (Sadler et al., 2022). Thermal heat sensitivity is measured using assays such as the hot plate, Hargreaves test, or tail-flick. The Hargreaves and tail-flick tests use radiant heat applied to the paw or tail, respectively, and the withdrawal threshold is recorded (Hargreaves et al., 1988). The hot plate test involves placing an animal on a heated metal plate, kept at the same constant high temperature (between 50–55 °C). Hind paw withdrawal, licking, number of paw flicks, and time to jump are common measurements used to assess thermal sensitivity in this assay (Deuis et al., 2017; Espejo and Mir, 1993). Mechanical sensitivity is typically assessed using von Frey filaments. In the von Frey assay, calibrated filaments of increasing force are applied to the area of interest, most often the hind paw, and the withdrawal response is recorded (Chaplan et al., 1994). Moreover, a common model of visceral pain is the acetic acid–induced writhing test, which measures abdominal constrictions (“writhes”) in response to acetic acid solution injection (Kuroyanagi et al., 2026; Sadler et al., 2022). These behavioral assays accurately detect the analgesic effects of clinically effective drugs, including opioids, nonsteroidal anti-inflammatory drugs (NSAIDs), and gabapentinoids (Gomez et al., 2023; Sadler et al., 2022). However, their clinical relevance has been questioned (Bradman et al., 2015; Treede et al., 2002). One concern is that these assays primarily measure reflexive and evoked-pain responses and may not adequately capture the emotional and cognitive dimensions of pain. It therefore remains unclear to what extent these behaviors reflect higher-order pain processing versus spinal reflex circuits (Treede et al., 2002). Another limitation is that patients with chronic pain most commonly report ongoing spontaneous pain or movement-evoked pain, rather than stimulus-evoked hypersensitivity (Ma et al., 2022; Sadler et al., 2022; Tappe-Theodor et al., 2019). To address these limitations, several behavioral paradigms have been developed that aim to better capture the ongoing, motivational, and affective components of pain. These include facial grimace scales, conditioned place preference paradigms using non-addictive analgesics to reveal relief of ongoing pain, and mechanical conflict avoidance assays, which assess the motivational cost of avoiding painful stimuli (Gaffney et al., 2022; Hung et al., 2015; Inyang et al., 2025; Langford et al., 2010).

A wide range of preclinical models have been developed to capture the heterogeneity of chronic pain conditions. Neuropathic pain is commonly induced by peripheral nerve injury models, such as the spared nerve injury (SNI) model (Decosterd and Woolf, 2000), or by systemic treatments that cause peripheral neuropathy, including chemotherapeutic agents like paclitaxel or platinum-based drugs (Inyang et al., 2025). Neuropathic pain can also be modeled in metabolic disease contexts, for example, using streptozotocin (STZ) to induce type 1 diabetes and diabetic neuropathy (Sullivan et al., 2007). Other models aim to reproduce different clinical pain conditions. Postoperative pain is typically modeled using surgical incision paradigms, such as paw incision (Cowie and Stucky, 2019; de Souza et al., 2026; Laumet et al., 2025). Inflammatory pain can be induced by the injection of agents such as complete Freund’s adjuvant (CFA) or formalin, which are widely used to model persistent and spontaneous pain resulting from neurogenic inflammatory mechanisms (Gregory et al., 2013; Sadler et al., 2022). In addition, migraine-like behaviors in rodents can be elicited through exposure to stressors that trigger migraine-relevant responses (Lackovic et al., 2021). Measurement of migraine-like behaviors includes assessing periorbital sensitivity with facial von Frey and facial grimacing after the stressors, two clinically relevant behaviors also prevalent in human migraineurs (Langford et al., 2010; Mason et al., 2021; Shrestha et al., 1996; Weissman-Fogel et al., 2003).

### Neural mechanisms underlying chronic pain hypersensitivity

Tissue injury or inflammation leads to increased sensitivity to thermal and mechanical stimuli, typically measured in animals as reductions in paw withdrawal thresholds in behavioral assays such as the Hargreaves, hot plate, and von Frey tests (Pinho-Ribeiro et al., 2017; de Souza et al., 2026; Sadler et al., 2022). The magnitude and duration of hypersensitivity depend on the type of injury; for example, in rodents, nerve injury models, such as SNI, produce robust and long-lasting hypersensitivity (Khan et al., 2024; Sadler et al., 2022). These behavioral changes are accompanied by profound alterations in neuronal physiology (Laumet et al., 2015). In rodent models and patients suffering from neuropathic pain, peripheral nociceptors become hyperexcitable and generate spontaneous activity (North et al., 2019; Laumet et al., 2020) while inhibitory interneurons in the spinal cord lose inhibitory efficacy due to changes in ion gradients and translational regulation (Engle et al., 2012; Ibuki et al., 1997; Laffray et al., 2012; Laumet, 2026). In parallel, glial cells including astrocytes and microglia become activated, as indicated by increased expression of GFAP and IBA1, respectively (Konnova et al., 2023; Xie et al., 2019; Xu et al., 2017). Dysfunction of descending inhibitory pain pathways has also been reported in chronic pain patients and rodent models (Drake et al., 2021; Ossipov et al., 2014; Tao et al., 2019). Together, these changes reflect a shift in pain-processing neural circuits in which nociception-promoting neurons become hyperactive, sensory neurons display spontaneous activity, and inhibitory mechanisms that normally suppress pain signaling become compromised.

### G protein-coupled receptor (GPCR) signaling in pain

The GPCR family has been an attractive target for pharmacological development. Pain was one of the first diseases to be treated by GPCR agonists. Indeed, opioid-based drugs, like morphine, that bind to GPCRs have been used since the era of Mesopotamia by Assyrians and described in ancient Egyptian papyruses as a medical treatment for pain (Buchanan et al., 2024). Both natural (morphine, opium) and synthetic (fentanyl, hydrocodone, tramadol) opioids are agonists for the GPCR mu-opioid receptor (MOR). Upon binding these ligands, MOR blocks the activity of adenylate cyclase to reduce the production of cAMP, inhibits the opening of voltage-gated calcium channels (which in neurons prevents the release of neurotransmitters), and opens K+ channels on the post-synaptic neurons to trigger hyperpolarization and prevent neuronal depolarization (Matthes et al., 1996; Lipp, 1991). Opioid analgesics mimic the actions of endogenous opioid peptides, including β-endorphin, enkephalins, dynorphins, and endomorphins, which are synthesized and released throughout pain-processing pathways to regulate nociceptive transmission by activating opioid receptors (Corder et al., 2018). The remarkable efficacy of these drugs in treating moderate to severe pain highlights the therapeutic potential of targeting GPCR signaling. Opioids are extremely efficient in preclinical models to reduce acute pain sensitivity in the hotplate, Hargreaves, and von Frey assays (Casarrubea et al., 2016; KuKanich et al., 2005; Lavigne et al., 1989). In clinical care, opioids attenuate acute pain efficiently but present significant side effects like constipation, addiction, and ultimately respiratory depression (Paul et al., 2021). In contrast, opioids are inefficient against neuropathic pain and have mixed effects in other chronic pain conditions (Busse et al., 2018; Von Korff, 2013).

Another more recent example of successful GPCR targeting for pain treatment is the calcitonin gene-related peptide (CGRP) receptor. The canonical CGRP receptor is a heteromeric complex composed of the Gαs-coupled calcitonin receptor-like receptor (CLR) and receptor activity-modifying protein 1 (RAMP1). CGRP signaling through this receptor plays a critical role in migraine pathophysiology (Edvinsson et al., 2018). Upon CGRP binding, the receptor activates adenylyl cyclase, increasing intracellular cAMP and engaging downstream signaling pathways (Edvinsson et al., 2018; Russell et al., 2014). Accordingly, major advances in migraine treatment have included therapies that inhibit CGRP signaling, including monoclonal antibodies targeting CGRP or its receptor and small-molecule CGRP receptor antagonists known as gepants (Garelja and Hay, 2022). These therapies provide an important example of the successful translation of peptide-GPCR biology into new analgesic treatments. However, a substantial proportion of patients still do not achieve adequate migraine relief, emphasizing the continued need to identify additional therapeutic targets (Bucklan and Ahmed, 2020; Garelja and Hay, 2022).

These limitations have prompted growing interest in identifying alternative GPCRs and ligands that regulate pain processing and could provide safer or more effective therapeutic strategies. Among the many peptide–GPCR systems implicated in nociceptive modulation, the neuropeptide neurotensin (Nts) has emerged as an important regulator of pain circuits.

## Neurotensin (Nts) signaling via neurotensin receptors

Nts was first isolated from the bovine hypothalamus (Carraway and Leeman, 1973; Carraway and Leeman, 1975) but it is now recognized that it is expressed by and secreted from subsets of neurons throughout the nervous system (Uhl et al., 1977b; Uhl et al., 1979). Nts is also produced by a variety of other cell types in peripheral tissue such as populations of neuroendocrine cells in the small intestine (Frigerio et al., 1977; Gilliam-Vigh et al., 2023). In the brain, Nts is usually co-expressed with a classical neurotransmitter like glutamate or GABA (Brown et al., 2019; Tschumi and Beckstead, 2019). Nts exerts direct actions as a neuropeptide binding to neurotensin receptors (NtsRs) expressed on other cells (discussed below) and as a neuromodulator of other neurotransmitter systems including the GABAergic, glutamatergic, cholinergic, and dopaminergic systems (Boules et al., 2013; St-Gelais et al., 2006). Compared to the rapid effects of classical neurotransmitters, Nts action is slower and longer lasting, as characteristic of neuropeptides (Ferraro et al., 2008; Kitabgi, 1989; Petkova-Kirova et al., 2008; Rakovska et al., 1998; Salio et al., 2006). Like classical neurotransmitter release, neuropeptide release is calcium-dependent, but unlike classical neurotransmitters, they are released from dense core vesicles throughout the neuron, not exclusively at the synapse (van den Pol, 2012). The precursor pro-neurotensin is processed by prohormone convertases, mainly PC2, to form the biologically active 13 amino acid Nts peptide (Kitabgi, 2006). Nts action is terminated by three Zn metalloendopeptidases, which degrade Nts after secretion by cleaving at the C-terminal hexapeptide sequence of Nts, which is required for receptor activation (Kitabgi, 2006). Differences in this degradation process caused by the abundance and activity of Zn metalloendopeptidases could modulate Nts longevity and ability to engage Nts receptors to mediate its function (Kitabgi, 2006).

Binding assays revealed that Nts binds to membrane preparations with varying affinity, suggesting the existence of Nts receptors with differing affinities for Nts (Uhl et al., 1977a). Neurotensin receptor 1 (NtsR1) was isolated from bovine brain membrane in 1988 and murine brain in 1989, then was cloned in 1990 (Mazella et al., 1989; Mills et al., 1988; Tanaka et al., 1990). NtsR1 is a membrane-bound Gq-coupled GPCR that binds Nts with high affinity (Slosky et al., 2020; Vita et al., 1993). NtsR1 is expressed in select subsets of neurons throughout the central nervous system and in numerous cell types throughout the gastrointestinal tract and other peripheral tissue. In the brain, NtsR1 is notably found in hypothalamic regions, the basal ganglia, substantia nigra, amygdala, septal regions, the VTA, and the PAG (Alexander and Leeman, 1998; Mitchell et al., 2009; Woodworth and Brown, 2018).

Neurotensin receptor 2 (NtsR2) was identified in 1996. Like NtsR1, NtsR2 is a GPCR but with lower binding affinity for Nts (Chalon et al., 1996). In the central nervous system, NtsR2 is predominantly expressed in astrocytes and endothelial cells (Kyriatzis et al., 2021; Nouel et al., 1997; Woodworth and Perez-Bonilla, 2018)​, and in the periphery it is expressed throughout the gastrointestinal tract and in adipocytes (Fu et al., 2025; Schulz et al., 2006). Intriguingly, it remains unclear which Gα subunit NtsR2 couples to, and if this differs between NtsR2 expressed within different cell types. NtsR2 is thought to signal primarily through heterotrimeric G proteins of the Gαq family, leading to PLCβ activation, IP_3_ and diacylglycerol production, intracellular Ca^2+^ mobilization, and protein kinase C (PKC) activation (Vincent et al., 1999). In addition to this canonical Gαq pathway, NtsR2 activation has also been reported to stimulate alternative signaling pathways involving cGMP production and activation of cGMP-dependent protein kinase G (PKG) (Shiva et al., 2026). PKG can reduce intracellular Ca^2+^ levels by enhancing sarcoplasmic reticulum Ca^2+^ uptake and inhibiting Ca^2+^ influx through L-type calcium channels, thereby attenuating CaMKII- and calcineurin-dependent signaling (Kamel et al., 2023). However, whether these alternative NTSR2 signaling pathways contribute to pain modulation remains unknown. There are also at least two non-GPCR neurotensin receptors. Neurotensin receptor 3, also known as sortilin, is a non-GPCR Nts receptor with a single transmembrane domain. NtsR3/Sortilin is localized mainly to intracellular membranes in the endoplasmic reticulum and the trans-Golgi network, with a smaller amount on the cell surface. It is involved in protein sorting and intracellular trafficking (Mazella, 2022; Navarro et al., 2002). Nts receptor 4, also referred to as SorLA, is also a single transmembrane domain receptor, but little is known about its function (Jacobsen et al., 2001).

Although originally studied for its hypotensive effect, Nts has since been recognized as an important regulator of several physiological processes, including feeding and body weight, drinking behavior, gut motility, body temperature, and has emerged as a potent modulator of pain and analgesia (Carraway and Leeman, 1973; Clineschmidt and McGuffin, 1977; Cooke et al., 2009; Hawkins et al., 1989; Levine et al., 1983; Nemeroff et al., 1977; Remaury et al., 2002; Thor and Rosell, 1986).

## Neurotensin’s role in analgesia

Intracerebroventricular and intracisternal administration of Nts to rodents has antinociceptive effects in acute pain assays including the tail-flick test, acid-induced writhing test, and hot plate test (Alexander and Leeman, 1998; Clineschmidt and McGuffin, 1977; Nemeroff et al., 1977; Osbahr et al., 1981). Nts-deficient mice showed increased nociception in response to visceral pain compared to wild-type mice, corroborating the pain-relieving properties of Nts (Gui et al., 2004). Some studies have even suggested that Nts-induced analgesia is opioid-independent, as Nts-mediated pain relief is not reversed by the MOR antagonist naloxone (Behbehani and Pert, 1984; Bredeloux et al., 2006). These studies demonstrate the analgesic potential of Nts, but whether this is driven by NtsR1, NtsR2, or both is not fully understood.

### Knockout mice to study the role of NtsRs in pain

Nts receptor knockout mice have been used to study the effects of Nts receptors on nociception. However, as reviewed below, findings from NtsR1 and NtsR2 knockout mice have produced conflicting results and have not clearly established the roles of the receptors in pain modulation. For example, NtsR1 knockout mice did not show differences in basal acute thermal pain responding via hot plate or tail immersion assay, and their intact thermal nociception has been interpreted as NtsR1 not being required for thermal nociception (Maeno et al., 2004). Yet, in other studies administering Nts agonists increased thermal response latency in wild-type mice but had no effect on latency in NtsR1 knockout mice, indicating that NtsR1 is involved in Nts-mediated pain relief in thermal pain (Mechanic et al., 2009; Pettibone et al., 2002). In a different pain assay, NtsR1 knockout mice exhibited decreased phenyl-p-benzoquinone-induced writhing upon administration of Nts (Remaury et al., 2002), suggesting that NtsR1 does not alleviate visceral pain. Likewise, there are multiple reports that central or peripheral administration of Nts does not change responding to acute thermal or visceral pain in NtsR1 knockout vs. wild-type mice (Maeno et al., 2004; Mechanic et al., 2009; Pettibone et al., 2002; Remaury et al., 2002). The finding that Nts reduces writhing in NtsR1 knockout mice points to a role for NtsR2 in pain that differs from NtsR1. This has led to the interpretation that NtsR1 is not the ‘antinociceptive’ receptor isoform and that the field should focus on NtsR2 in pain processing. In support of this, blocking NtsR2 translation using antisense oligodeoxynucleotides decreased acetic acid-induced writhing in mice (Dubuc et al., 1999a). Furthermore, while NtsR1 knockout mice showed no difference in hot plate responses compared to wild-type mice, NtsR2 knockout mice exhibited normal paw withdrawal responses but a significantly increased latency to jump (Maeno et al., 2004). This dissociation suggests that NtsR2 may preferentially regulate the motivational or behavioral escape response to painful stimuli rather than basal nociceptive processing (Barik et al., 2018). Conversely, NtsR2 knockout mice have reduced stress-induced analgesia in a cold-water swim stress model (Lafrance et al., 2010). Yet, both NtsR1 and NtsR2 knockout mice exhibit analgesia in a writhing test when treated with Nts agonists (Smith et al., 2012). These findings support the possibility that each receptor isoform can contribute to pain processing and that they are compensatory (Smith et al., 2012). Another study found that morphine receptor-mediated analgesia was reduced in NtsR1 knockout mice with formalin-induced pain, suggesting that NtsR1-mediated analgesia may interact with the mu opioid system (Roussy et al., 2010).

Taken together, these diverging results from NtsR knockout mice have not provided a clear or consistent understanding of how NtsR1 and/or NtsR2 contribute to pain processing. Why might this be? First, the variety of pain assays used in these experiments makes results across studies difficult to interpret and may have contributed to contradictory results across NtsR knockout mouse studies (Roussy et al., 2010). Furthermore, since multiple lines of NtsR1 and NtsR2 knockout mice have been generated and studied, differences in the genetic knockout approach or background strains could underlie the variance in pain effects observed in the literature (reviewed in Schroeder and Leinninger, 2018). In addition, the Nts system plays a role in development, making it possible that whole-body knockouts of components of this system could lead to developmental differences in these mice compared to wild-type mice (Hara et al., 1982). Finally, the diversity of neurotensin receptor agonists used across studies makes direct comparisons challenging. The pharmacological properties and roles of the different NtsR agonists are discussed below. Overall, while global receptor knockout lines and systemic pharmacological studies implicate Nts receptors in nociception, they do not clarify the respective roles of NtsR1 vs. NtsR2, nor have they revealed the sites and circuits via which the receptor isoforms contribute to Nts-mediated analgesia. Because receptors can have different functions depending on where they are expressed and via which cell type (excitatory or inhibitory neuron, glial cell, etc.) more targeted studies are needed to determine how Nts receptor signaling contributes to pain signaling in specific brain regions, cells, and circuits.

## Roles of the neurotensin system through the neural pain pathway

### Nts and NtsR signaling in the ascending pain pathway (DRG-Spinal cord-brain)

Nts is an important regulator of physiological and behavioral states, and the Nts system is therefore widely distributed throughout the nervous system, including regions not directly involved in pain processing (Hu et al., 2026). Given the scope of this review, however, we focus specifically on peripheral nervous system, brain regions, and neural pathways implicated in pain signaling and modulation Aaron, 2025. Nts-expressing neurons are present in both the DRG and spinal cord of multiple species (Chen et al., 2022; Kawarada et al., 2000; Reinecke et al., 1983; Stanzione and Zieglgansberger, 1983). Within the DRG, *Nts* is expressed by two major neuronal populations: ATF3+ and SST + neurons (Bhuiyan et al., 2024; Shi et al., 2014). SST + neurons comprise a subset of nociceptors and pruriceptors, whereas the ATF3+ population includes C-fiber sensory neurons involved in both nociceptive signaling and innocuous mechano-sensation (Bhuiyan et al., 2024; Xie et al., 2023). *Ntsr2* is expressed by several sensory neuron subtypes, including TRPM8+ cold-sensitive neurons and peptidergic nociceptors (Bhuiyan et al., 2024). To date, neither Nts nor its receptors have been detected in non-neuronal cells of the DRG (Bhuiyan et al., 2024).

In the spinal cord, Nts, and its receptors NtsR1 and NtsR2 are highly localized within the superficial laminae of the dorsal horn (Laminae I and II), which are the primary regions for processing pain and sensory information (Davis et al., 2026; Faull et al., 1989). Using immunohistochemical tracing, scientists found that NtsR2 in rodents is primarily localized to primary afferent fibers and superficial dorsal horns, providing a direct spinal pathway for pain modulation (Sarret et al., 2005; Tétreault et al., 2013). *In vivo* and *ex vivo* studies support that Nts has an inhibitory effect on DRG and ascending spinal cord dorsal horn neurons. For example, in DRG neurons cultured from guinea pigs, Nts treatment activates Cl- channels, causing an inward current (Kawarada et al., 2000). Spinal dorsal horn neurons cultured from mice treated with intrathecal Nts were also inhibited (Zhang et al., 2021). Moreover, intrathecal Nts treatment in naïve mice increased their latency to thermal pain and reduced spontaneous pain in the formalin test (Zhang et al., 2021). NtsR1 appears to be an important mediator of these effects and could be a peripheral target for analgesia. The NtsR1 allosteric modulator (SBI-810) was demonstrated to prevent the sensitization of DRG and spinal cord neurons seen after formalin injection. Moreover, SBI-810 reduced the frequency of action potentials in mouse and human DRG neurons and suppressed spinal excitatory transmission (Guo et al., 2025). Agonism of NtsR1 has shown efficacy in models of postoperative pain, inflammatory pain, and neuropathic pain, without the negative side effects associated with opioids (Guo et al., 2025). This exciting work demonstrates that modulating peripheral NtsR1 signaling can have beneficial effects on acute and chronic pain. Additionally, NtsR2 agonists injected intrathecally alleviated acute and chronic pain in rodent models (Sarret et al., 2005; Tétreault et al., 2013). These studies support that Nts contributes to analgesia via inhibitory modulation of DRG and spinal cord dorsal horn neurons.

The spinothalamic tract conveys nociceptive input to the thalamus. The thalamus serves as a major relay for ascending nociceptive signals to cortical regions (Figure 1). The importance of the thalamus in pain processing is highlighted in imaging and electrophysiology studies showing that thalamic neurons are activated after noxious stimuli and that direct stimulation of the thalamus can cause pain responses (Friebel et al., 2011; Ohara and Lenz, 2003; Yen and Lu, 2013; Yen and Shaw, 2003). From the thalamus, central processing occurs and relays information to different parts of the cortex. Cortical regions contribute to pain processing. Viral tracing studies revealed that *Nts*-expressing DRG neurons, through multiple synapses, convey signals to the primary sensory cortex (Chen et al., 2022). The neuronal projections to the somatosensory cortex are necessary for localized perception of pain (Terrier et al., 2022). Furthermore, it is theorized that functional reorganization of the somatosensory cortex can contribute to chronic pain, as imaging studies have shown differences between brain activity in patients with versus without pain (Li et al., 2022). Alternatively, other ascending pathways like the spinoparabrachial tract provides nociceptive information to the amygdala processing the emotional aspect of pain (Basbaum et al., 2009). The central amygdala is important for both receiving input via the spinoparabrachial tract and projecting information to areas such as the LHA and PAG (Bernard et al., 1996; Li and Sheets, 2018). Notably, the central amygdala contains many neuropeptides related to pain behaviors, including Nts (Honkaniemi et al., 1990; Neugebauer et al., 2020). Neuroplasticity changes in the central amygdala are also connected to the transition from acute to chronic pain (Kiritoshi et al., 2024).

### Nts and NtsR signaling in the descending pain pathway (brain-spinal cord)

The periaqueductal gray (PAG) has long been a target of pain research, with scientists identifying that electrically stimulating the area is effective in promoting analgesia as far back as 1969 (Barbaro, 1988; Mayer et al., 1971; Reynolds, 1969). This area is important for the modulation of pain signaling, as descending projections from the PAG to the spinal dorsal horn regulate ascending inputs via inhibitory signals (Heinricher et al., 2009; Wang et al., 2022). It is now understood that the PAG contains endogenous opioids and opioid receptors to mediate nociceptive inhibition (Adams, 1976; Akil et al., 1976; Barbaro, 1988; Vaughan et al., 1997). The activation of the descending inhibitory pain pathway occurs when opioidergic neurons disinhibit excitatory projections between the medial prefrontal cortex (mPFC) and the PAG. The opioid receptor signaling in the mPFC silences GABAergic interneurons and thus allows the descending inhibitory pathway in the PAG to become active and induce analgesia (Figure 1) (Neyama et al., 2025). While the PAG is well known for its role in descending antinociceptive signaling, it can also facilitate pain depending on the neuronal populations engaged. Studies using transgenic mice demonstrated that activation of glutamatergic PAG neurons reduces nociception, whereas activation of GABAergic PAG neurons promotes pain hypersensitivity (Samineni et al., 2017). Also vital to the descending pain pathway is the dorsal raphe nucleus (DRN), located ventromedial to the PAG and rich with serotonin (5-HT), GABAergic, dopamine (DA), and glutamatergic neurons (Huang et al., 2019; Zhang et al., 2024). Specifically, the DRN is thought to inhibit nociceptive signaling via these neurotransmitters, such as DRN serotonergic neurons projecting to the SDH where 5-HT inhibits pain transmission, inducing antinociceptive behavioral responses (Ossipov et al., 2014). Another midbrain region recently investigated in the context of pain transmission is the interpeduncular nucleus (IPN). Traditionally viewed as being involved with stress, reward, anxiety, and depression behaviors, neuroanatomical studies showing projections from the IPN to pain-associated areas such as the LHA, VTA, and DRN have raised the possibility that the IPN may also be involved in pain (Ables et al., 2023; McLaughlin et al., 2017; Sherafat et al., 2020; Song et al., 2026; Xu et al., 2018). A recent study showed that GABAergic projections from the IPN to the DRN mediated pain and depression comorbidity after CFA injection, highlighting the role this region may play in pain (Song et al., 2026).

The PAG sends projections to the rostral ventromedial medulla (RVM), another area rich in endogenous opioids and opioid receptors, and the RVM in turn projects to the spinal dorsal horn (Figure 1) (Bagley and Ingram, 2020; Heinricher et al., 1992). The RVM was once thought to be an ‘analgesia center’ because stimulating it has antinociceptive effects, but this region can also play a role in promoting pain (De Preter and Heinricher, 2024). The modulation of pain in the RVM is controlled primarily through ‘ON’ and ‘OFF’ cells that fire in response to nociceptive input (Heinricher et al., 1992; Heinricher and Neubert, 2004; Heinricher, 2016) and project to the spinal dorsal horn. These RVM cells differ in their response to nociceptive input, with noxious heat and mechanical stimulation causing ‘ON’ cells to fire and ‘OFF’ cells to pause before withdrawal (Fields et al., 1991; Fields and Heinricher, 1985). Persistent firing of RVM ‘ON’ cells has been shown to precede hyperalgesia and pharmacologically inhibiting these cells reduces hyperalgesia (Martenson et al., 2009). Opioids acting in the RVM suppress ‘ON’ cells and disinhibit ‘OFF’ cells, leading to analgesic effects (Bagley and Ingram, 2020; Heinricher and Neubert, 2004; Morgan et al., 1992; Neubert et al., 2004).

Several structures of the descending pain pathway contain Nts receptor-expressing cells that could play a role in pain signaling, including the PAG and RVM (Mitchell et al., 2009). Nts receptors are also expressed by cells in brain regions that are interconnected with the PAG, such as the LHA and the VTA (Khan et al., 2024; Woodworth et al., 2017). Understanding how Nts receptors function within regions that either constitute or engage the descending pain modulatory pathway will provide important insight into the neural circuits underlying Nts-mediated analgesia. Local microinjection of Nts into the PAG results in analgesia in acute pain assays (Kalivas et al., 1982; Mitchell et al., 2009). The PAG is innervated by Nts-expressing neurons originating in the BNST, amygdala, and the LHA (Behbehani et al., 1988; Gray and Magnuson, 1992). Based on this, endogenous release of Nts from these regions to the PAG could be a mechanism to modulate pain. Indeed, Nts protein levels and Nts release are increased in rodent pain models, which may be an attempt to increase endogenous signaling to Nts receptors to mediate pain relief. The PAG contains cells expressing Nts, NtsR1, and NtsR2 (Al-Rodhan, 1991; Asselin et al., 2001; Mitchell et al., 2009; Sarret et al., 2003; Shipley, McLean and Behbehani, 1987; Williams and Beitz, 1993; Williams et al., 1995). NtsR2 has been shown to be upregulated during neuroinflammation, so it is possible that PAG astrocytic expression of NtsR2 is increased in painful states, but this has yet to be studied (Kyriatzis et al., 2021). The specific roles of NtsRs within the PAG remains unclear. One study showed that Nts signaling in the PAG was blocked by both a NtsR1-specific antagonist and a Nts receptor pan-antagonist, suggesting that this signaling takes place through NtsR1, with the possibility of NtsR2 playing a role as well (Mitchell et al., 2009). However, another study found that Nts-evoked PAG signaling to the RVM was not blocked by NtsR1 or NtsR2 antagonists injected into the PAG and that this signaling was G_q/11_-dependent, suggesting the involvement of a distinct receptor (Li et al., 2001). A challenge in these studies is the reliance on NtsR antagonists and intracellular responses to characterize Nts and NtsR-specific responses, but which cannot verify that the measured cells indeed express specific NtsR isoforms (or not). In the absence of such confirmation, it is difficult to discern if cellular responses are due to direct or indirect Nts/NtsR signaling. In the future, it will be important to use approaches that identify specific NtsR-expressing neurons in the PAG to reveal if/how they respond to Nts, and their contributions to pain processing.

While the exact NtsR isoforms in the PAG that modulate pain processing remain unclear, there is evidence pointing to circuit mechanisms for Nts-mediated analgesia. The most notable projection of the PAG in the descending pain pathway is to the RVM where it is known to release Nts (Davis et al, 2026; Wang et al., 2014). Indeed, Nts signaling in the PAG is typically associated with excitation of RVM-projecting neurons that inhibit pain transmission. The RVM is characterized by containing ON-cells that facilitate nociception, OFF-cells that inhibit it, and neutral cells that don’t respond to pain (Peng et al., 2023). The activity of these subsets of neurons has a major impact on descending pain signaling. Injection of Nts into the RVM produces different effects on nociception depending on dose. Injection of a low dose produces hyperalgesia (increased sensitivity to pain), likely by activating ON-cells (Neubert et al., 2004; Urban and Smith, 1993). Meanwhile, studies have found that a high dose of Nts in the PAG either has no effect on nociception or has an analgesic effect, presumably via activating neutral cells or OFF-cells respectively (Neubert et al., 2004; Urban and Smith, 1993). Both NtsR1- and NtsR2- expressing neurons are found in the RVM (Buhler et al., 2005). While NtsR1 neurons in the RVM largely co-express with serotonin, NtsR2 neurons rarely do (Buhler et al., 2005). The majority of serotonergic RVM neurons are neutral cells, followed by OFF-cells, and then ON-cells (Potrebic et al., 1995). It follows that there are more NtsR1-expressing OFF-cells that inhibit pain transmission than ON-cells that facilitate it. One theory for the dose-dependent response is that a low dose will bind to the high-affinity NtsR1 and activate ON-cells, but a high dose will activate OFF-cells to a stronger degree (Roussy et al., 2008). The distribution of NtsR2 neurons among neutral cells, OFF-cells, and ON-cells of the RVM is still unknown. Studies also support an indirect circuit mechanism by which Nts modulates pain via the PAG, resulting in the disinhibition of descending pain signaling. Slice electrophysiology showed that Nts in the PAG activates glutamatergic neurons, inducing the release of endocannabinoids that suppress local GABAergic signaling, resulting in disinhibition of descending analgesic signaling, including to the RVM (Mitchell et al., 2009). Hence, Nts release can produce different effects depending on the connectivity of the postsynaptic neuron, Together, these electrophysiological studies provide strong evidence that Nts in the PAG coordinates pain signaling via direct and indirect circuits, but there is a need for *in vivo* studies to corroborate these findings with antinociceptive behavioral responses.

Just adjacent to the PAG lies the dorsal raphe nucleus (DRN), which has also recently been implicated in Nts-mediated analgesia. The DRN is best-known for production of serotonin. The DRN projects to key areas involved in the descending pain (PAG, RVM, and spinal cord) and acts as a serotonergic regulator (Qing-Ping and Nakai, 1994). It notably receives co-released Nts and GABA from the interpeduncular nucleus (IPN), and this projection is associated with inducing pain and depression-like behavior (Song et al., 2026). Optogenetic and chemogenetic activation of this IPN to DRN projection increased mechanical pain sensitivity in naïve mice, while inhibition alleviated inflammatory-induced mechanical pain hypersensitivity (Song et al., 2026). Chemogenetic activation of IPN Nts-expressing projections to the DRN, combined with local pharmacological antagonism of DRN Nts receptors, did not alter pain responses, whereas GABA receptor antagonism reduced pain sensitivity (Song et al., 2026). These findings suggest that GABA release from these Nts-expressing projections, rather than Nts itself, is necessary for modulating pain signaling within this pathway. The role of Nts release from the IPN Nts-expressing neurons to the DRN remains unclear. However, Nts receptor antagonism without chemogenetic activation of this pathway led to a significant decrease in mechanical pain (Song et al., 2026), suggesting that Nts signaling in the DRN is important for analgesic signaling independent of the IPN-to-DRN pathway.

### Other brain sites via which Nts-NtsRs may modulate pain processing

An abundant source of endogenous Nts in the brain is the lateral hypothalamic area (LHA) (Khan et al., 2024; Schroeder et al., 2019; Zahm et al., 2001). The LHA is also a site of pain modulation, as some LHA neurons respond to noxious stimuli and LHA stimulation promotes analgesia (Aimone et al., 1988; Dafny et al., 1996; Lopez et al., 1991). GABA released from the lateral septum to the LHA is increased during von Frey and open field testing, suggesting an important role of this circuit in processing nociceptive and anxiolytic stimuli (Wang et al., 2023). The expression of Nts in the LHA, as assessed by RNAscope, increases in response to CFA or nerve injury in mice (Khan et al., 2024). Moreover, chemogenetic activation of Nts-expressing LHA neurons in mice alleviates mechanical sensitivity in neuropathic pain and inflammatory pain models in both sexes (Khan et al., 2024). Furthermore, these analgesic effects were reversed by a neurotensin receptor pan-antagonist SR142948, supporting a role for Nts signaling in this physiological effect. Intriguingly, no changes in thermal pain response were observed, suggesting that Nts neurons in the LHA are only involved in specific modalities of pain. The LHA plays a central role in sensing and integrating physiological, metabolic, and homeostatic signals. Therefore, the LHA may have a critical role in coordinating metabolic dysregulation and pain, such as comorbid obesity and chronic pain (Khan et al., 2024). Nts-expressing neurons in the LHA project to several brain regions involved in pain processing such as the PAG, BNST, parabrachial nucleus, and VTA (Kurt et al., 2022). The contributions of these specific projections to Nts-mediated analgesia remain unclear, as well as whether it requires NtsR1 and/or NtsR2.

The VTA is another limbic region that plays a role in the emotional aspect of pain and pain sensitivity. Thalamic, cortical, and hindbrain connections converge and integrate in the VTA to modulate affective feelings and behaviors (Beier et al., 2015; Faget et al., 2016; Markovic et al., 2021; Zhang et al., 2025). This occurs, in part, via the large population of dopamine neurons in the VTA that modulate reward behaviors, addiction, and depression, and therefore may contribute to affective pain associated with these states (Flores-García et al., 2023). Indeed, the VTA has been suggested to be the connection between chronic pain and depression (Zhang et al., 2025). While the VTA is primarily comprised of dopamine neurons, it also contains smaller populations of GABAergic and glutamatergic neurons (Flores-García et al., 2023). GABAergic neurons project locally onto dopamine neurons, and so may modulate dopamine-mediated functions including affective aspects of pain and pain sensitivity (Sato et al., 2022; Wang et al., 2025). Furthermore, activating glutamatergic VTA neurons that project to the nucleus accumbens increases pain and pain-induced anxiety, while inhibiting this pathway can stimulate relief for both acute and chronic pain (Abdul et al., 2022).

Another brain region that receives Nts input is the bed nucleus of the stria terminalis (BNST). BNST neurons project to the VTA and suppress dopamine signaling as well as increase inhibitory signaling to the LHA, which collectively increases anxiety-like behaviors (Takahashi et al., 2019; Yamauchi et al., 2022). Nociceptive inputs from the spinal dorsal horn, ventromedial hypothalamus, and amygdala also converge in the BNST, suggesting the area plays a role in the emotional components of pain (Braz et al., 2005; Minami, 2009). The importance of the BNST in the affective aspect, but not sensory component, of pain is demonstrated in an experiment in which the BNST is ablated with ibotenic acid. Compared to sham mice, mice with a lesioned BNST spent more time in the ‘pain-paired’ side of the chamber during the conditioned place aversion assay but did not exhibit a difference in peripheral nociceptive behaviors after acetic acid or formalin injection (Deyama et al., 2007). In particular, the NtsR1-expressing neurons in the oval division of the BNST both receive input from and send output to several brain regions implicated in pain processing that express Nts and its receptors such as the PAG, LHA, and parabrachial nucleus (Lu et al., 2024). Optogenetic activation of NtsR1-expressing neurons in the oval division of the BNST demonstrates a mild analgesic effect in mice by decreasing CFA-induced mechanical sensitivity and thermal sensitivity but did not alleviate pain from a formalin injection into the whisker pad. Additionally, it suppresses anxiety-like behavior as measured by open field and elevated plus maze and decreases heart rate (Lu et al., 2024). This could suggest that Nts signaling in the BNST is responsible for emotional responses to pain.

### Neurotensin at the neuroimmune interface in migraine and other painful conditions

Migraine is one of the most common and disabling pain conditions among individuals under the age of 50 in the United States (Ashina, 2020; Cohen, 2024; Steiner et al., 2016). Although the precise causes of migraine remain incompletely understood, hyperactivation of TG primary afferents, including nociceptors, and the associated release of neuropeptides are considered key mechanisms underlying migraine pathophysiology. The release of these neuropeptides triggers neurogenic inflammation, characterized by mast cell degranulation and vasodilation (Conti et al., 2019; Gürkan et al., 2000; Salahi et al., 2022). Electrical stimulation of the TG has been shown to induce the release of Nts in the caudal trigeminal nucleus, suggesting a potential role for this neuropeptide in migraine mechanisms (Samsam et al., 2002). Alternatively, Nts can also be released by meningeal lymphatic vessels (Schmidt et al., 2025), where it may activate meningeal mast cells. Upon degranulation, mast cells release a broad range of inflammatory mediators, including tumor necrosis factor (TNF), interleukin (IL)-6, and serotonin (Conti et al., 2019; Levy, 2012; Moretti et al., 2014; Theoharides et al., 2010). These mediators sensitize and activate TG neurons, thereby amplifying nociceptive signaling within the trigeminovascular system (Figure 2). Nts has been shown to induce mast cell degranulation through activation of NtsR1, which is expressed on mast cells. Pharmacological blockade of this receptor with the antagonist SR48692 prevents Nts-induced mast cell degranulation (Carraway et al., 1982; Miller et al., 1998; Piliponsky et al., 2008). Interestingly, mast cells can also degrade Nts itself, suggesting a complex and bidirectional regulatory relationship between mast cells and Nts signaling (Piliponsky et al., 2008). Despite these observations, the precise contributions of both mast cells and Nts to migraine pathophysiology remain incompletely defined. Further research is required to determine whether targeting Nts signaling may offer novel therapeutic opportunities for migraine treatment. It is interesting to note that the role of Nts in migraine, which is presumably pronociceptive, seems to contrast with the antinociceptive effect of Nts on somatic pain.

**FIGURE 2:**
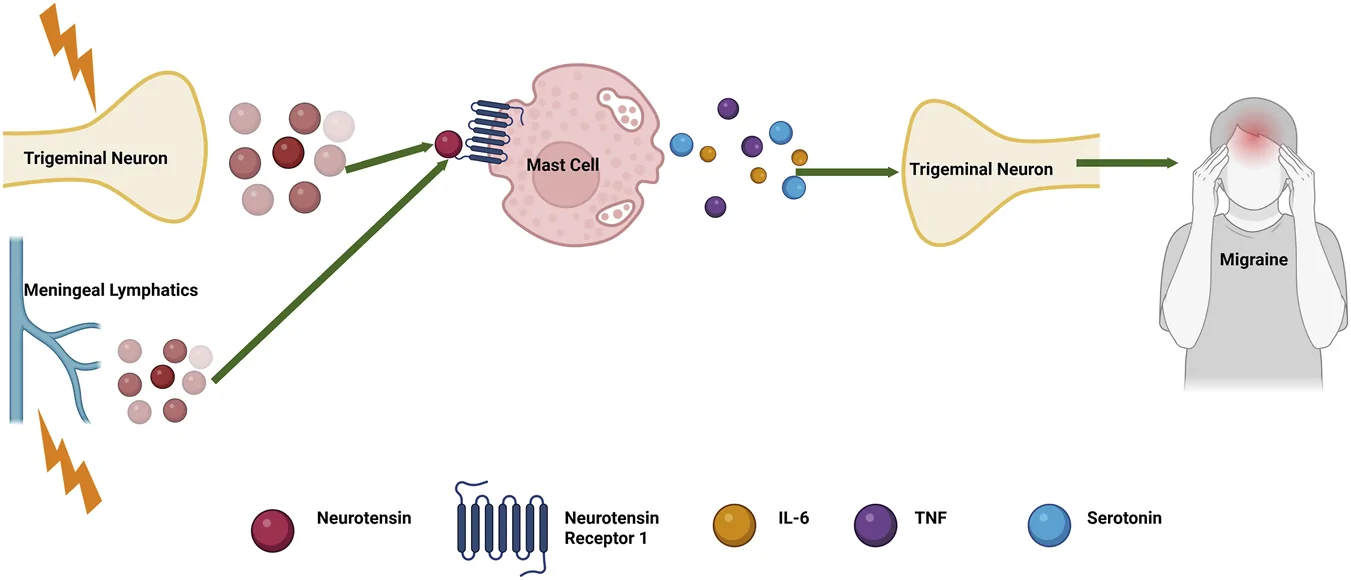
Neurotensin signaling within pain circuits and neuroimmune interactions. Following stimulation, Nts (red) is released from TG neurons and/or within the meninges. Nts may act on mast cells through NtsR1, promoting mast cell degranulation and the release of inflammatory mediators, including tumor necrosis factor (TNF), interleukin-6 (IL-6), and serotonin. These mediators may subsequently sensitize TG neurons, potentially contributing to migraine pathophysiology.

Beyond migraine, neurotensin–mast cell interactions may also be relevant to other chronic pain conditions, including fibromyalgia and endometriosis. Fibromyalgia is characterized by widespread chronic pain and has been associated with neuroinflammatory mechanisms and increased numbers of mast cells in the skin (Theoharides et al., 2015; Theoharides et al., 2019). Neurotensin has been proposed as one of several neuropeptides capable of stimulating mast cells (Carraway et al., 1982; Feldberg et al., 1998; Theoharides et al., 2012), which subsequently release inflammatory and sensitizing mediators that can activate peripheral sensory neurons and potentially sustain pain (Mai et al., 2021). However, the available evidence linking neurotensin itself to fibromyalgia remains limited, and reported alterations in neurotensin levels in patients have not been consistent, leaving its contribution to fibromyalgia pathophysiology unclear (Navarro-Gonzalez et al., 2025; Tsilioni et al., 2016).

Endometriosis represents another potential context in which neurotensin–mast cell signaling could contribute to pain. Endometriosis is an estrogen-dependent inflammatory disease frequently associated with severe chronic pelvic pain, and estrogen is considered a major trophic factor driving disease development (Chantalat et al., 2020; Wang et al., 2025). Endometriotic lesions contain increased numbers of mast cells, and elevated mast cell tryptase has been reported in the peritoneal fluid of patients (Golinska et al., 2025; McCallion et al., 2022). Moreover, estradiol can promote mast cell maturation and degranulation, while experimental estrogen treatment further increases mast cell accumulation within endometriotic lesions (Makoui et al., 2025; Wang et al., 2025). Because estrogen can regulate neurotensin expression in other biological contexts (Alexander and Leeman, 1994; Watters and Dorsa, 1998), these observations raise the possibility that neurotensin could participate in estrogen-dependent neuroimmune signaling in endometriosis. However, a direct role for neurotensin in endometriosis-associated pain has not yet been established and represents an interesting area for future investigation.

## Potential pharmacological modulation of NtsRs for analgesia

Modulation of the Nts system shows great potential as a drug target for the treatment of multiple types of pain. Nts-induced analgesia is opioid receptor-independent and even has additive effects with morphine (Boules et al., 2009). This suggests that Nts could replace opioids or be used as a complementary treatment to allow for a lower dose of opioids. Unlike opioids, Nts is not known to cause addiction and respiratory depression. Indeed, in some cases Nts receptor agonists even decrease addictive behaviors and psychostimulant use in mice (Slosky et al., 2020). However, Nts does have characteristics that make its use as a pain treatment clinically challenging. Nts elicits negative side effects, specifically linked to NtsR1, such as hypotension and hypothermia (Bissette et al., 1976; Carraway and Leeman, 1973). In addition, native Nts is quickly degraded in the bloodstream and does not cross the blood-brain barrier, limiting access to the brain (Demeule et al., 2014; Li et al., 2020). Much effort has been put into developing Nts receptor modulators that produce analgesia while overcoming these challenges.

Several Nts receptor agonists have been developed with enhanced stability compared to endogenous Nts (summarized in Table 1). NT69L is a Nts receptor agonist that binds to both NtsR1 and NtsR2 (Boules et al., 2009; Mechanic et al., 2009; Roussy et al., 2009). Rats treated with NT69L had longer latency to respond in the hot plate test than saline-injected controls. NT69L also reduced formalin-induced nociceptive behavior following intrathecal administration, demonstrating efficacy in a neurogenic inflammatory pain model, while also triggering hypothermia and sedation. Similarly, in mice, NT69L and NT-2, another Nts receptor agonist, both decreased nociceptive response in the tail immersion test for thermal pain. However, these experiments were performed in pain-free animals, so the effect of these drugs in rodent pain models is unclear. Antinociceptive doses of NT69L and NT-2 also caused a significant decrease in body temperature in mice. NT69L also has antinociceptive effects in rhesus monkeys (Fantegrossi et al., 2005). After injecting capsaicin into the tail to induce pain, monkeys underwent a warm water tail-withdrawal test to assess thermal pain sensitivity. Intravenous administration of NT69L increased latency, supporting the results found in rats that NT69L decreases response to thermal stimuli. NT69L had adverse effects on blood pressure and body temperature, however, which prevented evaluation of doses of over 0.03 mg/kg. The same study also investigated NT69L’s reinforcing abilities by performing a self-administration assay. Monkeys did not increase lever pressing to earn NT69L infusion, suggesting that this drug has low abuse potential. This is a major advantage over opioid-based painkillers, which are known to have a high potential for abuse (Dowell et al., 2022; Volkow and McLellan, 2016). More recently, additional Nts receptor agonists have been developed and tested across pain models. ABS212 decreased acetic acid-induced writhing, thermal pain response in the hot plate and tail flick test, pain responses due to formalin injection, and even mechanical hypersensitivity in a model of neuropathic pain in rats. This drug showed a comprehensive antinociceptive effect across numerous pain models but also significantly decreased body temperature (Hughes et al., 2010). Similarly, An2-NT (8-13) decreased formalin-induced pain behavior in rats, but effects on body temperature and blood pressure were not recorded (Eiselt et al., 2019b).

**TABLE 1:** Effect of Nts receptor agonists across pain models and assays in rodents.

Agonist	Animal	Route	Dose	Model	Pain	Side effects	References
NtsR pan-agonists							
An2-NT (8-13)	*Rattus norvegi-cus*	IV	0.018 mg/kg	Formalin	Formalin test ↓	Hypotension	Eiselt et al., 2019b https://doi.org/10.1016/j.ejphar.2019.01.048
NT-2	*Mus muscul-us*	IP	1.0 mg/kg	Naïve	Tail immersion ↓	Hypothermia Sedation	Mechanic et al., 2009 10.1016/j.euroneuro.2009.01.004
NT69L	*Mus muscul-us* *Rattus norvegi-cus* *Macaca mulatta*	IP IP IT IV	3.0 mg/kg 0.25-1 mg/kg 5 μg/kg 0.01-0.03 mg/kg	Naïve Naïve Formalin Capsaicin	Tail immersion ↓ Hot plate ↓ Formalin test ↓ Tail immersion	Hypothermia Sedation Modest hypothermia Hypotension	Boules et al., 2009 10.1016/j.brainres.2009.07.086 Fantegrossi et al., 2005 10.1016/j.pbb.2004.12.005 Mechanic et al., 2009 10.1016/j.euroneuro.2009.01.004 Roussy et al., 2009 10.1186/1744-8069-5-38
ABS212	*Rattus norvegi-cus*	IP	10 mg/kg	Naïve Formalin Chung model	Hot plate ↓ Acetic acid writhing test ↓ Tail-flick test ↓ Formalin test ↓ von Frey ↓	Hypothermia	Hughes et al., 2010 10.1021/jm100092s
CGX	*Rattus norvegi-cus* *Mus muscul-us*	IT	0.02-0.5 nmol 0.5 nmol	Paw incision SNL Paw incision pSNL CIBP	von Frey ↓ Hargreaves ↓ von Frey ↓ Hargreaves ↓ von Frey ↓ von Frey ↓ von Frey ↓ Spontaneous flinching ↓	No motor impairment or tolerance	Martin et al., 2025 10.1097/j.pain.0000000000003391 Martin et al., 2022 10.1097/j.pain.0000000000002561
NtsR1 agonists							
PD 149163	*Rattus norvegi-cus*	IP IT	0.1, 0.3 mg/kg 10, 30 ng	CIPN CIPN	von Frey ↓ von Frey ↓	​	Yin et al., 2020 10.3344/kjp.2020.33.4.318
NtsR1 allosteric modulators							
SBI-810 and SBI-553 SBI-810 SBI-810 SBI-810 SBI-810	*Mus muscul-us*	IP IT	12 mg/kg 22 nmol	Naïve Paw incision Bone fracture SNI Formalin Naïve SNI	von Frey ↓ Hot plate ↓ Hargreaves ↓ Dry ice test ↓ Randall-Selitto paw pressure test ↓ von Frey ↓ Guarding behavior ↓ Grimace score ↓ von Frey ↓ Guarding behavior ↓ Acetone test ↓ von Frey ↓ Acetone test ↓ Formalin test ↓ von Frey ↓ von Frey ↓	No motor, blood flow, or tactile sensation impairment	Guo et al., 2025 https://doi.org/10.1016/j.cell.2025.04.038
NtsR2 agonists							
Levocabastine	*Mus muscul-us* *Rattus norvegi-cus*	ICV IT	50-1350 pmol 600-2400 pmol 5 μg/kg 5 μg/kg	Naïve Naïve Formalin CCI	Hotplate = Acetic acid writhing test ↓ Tail-flick test ↓ Formalin test ↓ von Frey ↓ Weight bearing ↑	No significant effect on body temperature	Dubuc et al., 1999b https://doi.org/10.1016/S0014-2999(99)00554-3 Roussy et al., 2009 10.1186/1744-8069-5-38 Sarret et al., 2005 10.1523/JNEUROSCI.0810-05.2005 Tétreault et al., 2013 https://doi.org/10.1096/fj.12-225540
NT79	*Rattus norvegi-cus*	IP	5 mg/kg	Naïve	Hot plate = Acetic acid writhing test ↓	No significant effect on body temperature or blood pressure	Boules et al., 2009 10.1016/j.brainres.2009.10.050
Compound 13	*Rattus norvegi-cus*	IT	2-62 nmol	Formalin	Formalin test ↓	No significant effect on body temperature or blood pressure	Eiselt et al., 2019a 10.1021/acschemneuro.9b00390
CR-01–64	*Rattus norvegi-cus*	IT	3-60 μg/kg	Naïve Formalin CFA	Tail-flick test ↓ Formalin test ↓ von Frey ↓	No significant effect on body temperature Modest hypotension	Chartier et al., 2021 10.1016/j.biopha.2021.111861
JMV5296	*Rattus norvegi-cus*	IT	60 μg/kg	Naïve Formalin CFA	Tail-flick test ↓ Formalin test ↓ von Frey ↓	No significant effect on body temperature Modest hypotension	Vivancos et al., 2021 10.1016/j.bbr.2021.113189
JMV431	*Rattus norvegi-cus*	IT	12-48 nmol 5-60 μg/kg 3-90 μg/kg	Naïve Formalin CCI	Tail-flick test ↓ Formalin test ↓ von Frey ↓ Weight bearing ↑	​	Roussy et al., 2009 10.1186/1744-8069-5-38 Sarret et al., 2005 10.1523/JNEUROSCI.0810-05.2005 Tétreault et al., 2013 https://doi.org/10.1096/fj.12-225540
β-lactotensin	*Rattus norvegi-cus*	IT	180 μg/kg	CCI	von Frey ↓ Weight bearing =	​	Tétreault et al., 2013 https://doi.org/10.1096/fj.12-225540

Summary of neurotensin receptor agonists evaluated in preclinical studies including route of administration (intraperitoneal (IP) or intrathecal (IT)), pain model (Model), behavioral assay used and pain response (Assay and Pain Response), and the supporting references. For degree of pain response, = denotes no change in pain and ↓ denotes a decrease in pain. CCI: chronic constriction injury, CIBP: cancer-induced bone pain, CIPN: chemotherapy induced peripheral neuropathy, SNI: spared nerve injury. SNL: sciatic nerve ligation, pSNL: partial sciatic nerve ligation.

NtsR1-specific agonists have also been developed and studied for their antinociceptive effects. The NtsR1 agonist PD 149163 successfully crosses the blood-brain barrier and induces analgesia (Feifel et al., 2010; Xue et al., 2017; Yin et al., 2020). PD 149163 administered at a dose of 0.5 mg/kg reduces cisplatin-induced peripheral neuropathic pain in rats (Yin et al., 2020). Both intrathecal and intraperitoneal administration of PD 149163 decreased mechanical sensitivity as measured by von Frey filaments. However, other studies found that doses of 0.25, 0.5, and 1.0 mg/kg produced hypothermia in rats (Feifel et al., 2010; Xue et al., 2017). Together, these studies show that Nts receptor pan-agonists and NtsR1 agonists are effective in producing analgesia but are limited by adverse side effects that occur at the same doses necessary for pain relief.

One method to combat this is to modulate Nts receptors instead of broadly activating them. The most promising preclinical analgesic drugs acting on NtsR1 are SBI-553 and SBI-810. Both are β-arrestin-biased allosteric modulators of NtsR1 capable of binding to a non-active site on NtsR1 to induce β-arrestin signaling, but not Gq signaling, which is associated with negative side effects (Guo et al., 2025). SBI-553 and SBI-810 are similar in structure and efficacy. SBI-810 successfully alleviated both acute and chronic pain in a comprehensive array of pain models in mice without impairing motor function, or blood flow (Guo et al., 2025). SBI-810’s antinociceptive effects come mainly from binding to NtsR1 in the DRG and spinal cord to block pain signaling. In the DRG, SBI-810 decreases ERK phosphorylation, a marker of neuronal activation, and surface expression of Nav1.7, a key voltage-gated sodium channel in pain signaling. Strikingly, SBI-810 also reduced neuronal excitability in mouse and human DRGs. In the spinal cord, it decreases ERK phosphorylation and NMDA receptor signaling and associated excitatory postsynaptic currents. Together, these effects dampen nociceptive transmission. Moreover, SBI-810 reduced opioid-induced constipation and addiction in mouse models. The potential role of SBI 553/810 for analgesia via the brain has yet to be examined. However, given the abundance of NtsR1 in brain areas that modulate pain it is possible that SBI 553/810 could also contribute to analgesia via central mechanisms. Hence, SBI-810 represents a promising candidate analgesic that may avoid several drawbacks associated with opioids and earlier Nts receptor agonists, supporting further development of biased allosteric modulators.

Another method to avoid hypothermic and hypotensive effects may be to selectively target NtsR2. While knockout and knockdown mouse studies suggested NtsR1 is involved in Nts-mediated analgesia, NtsR2 appears to be more involved in pain modulation (Dubuc et al., 1999a; Mechanic et al., 2009; Pettibone et al., 2002; Remaury et al., 2002). Contulakin-G (CGX), a conotoxin with sequence homology to Nts, provides strong evidence that spinal NtsR2 activation can mediate analgesia. Intrathecal administration of CGX reduces thermal and mechanical hypersensitivity in postoperative pain models and flinching behavior in a cancer-induced bone pain model, with effects observed in both mice and rats and without apparent sex differences. Although CGX binds both NtsR1 and NtsR2, spinal NtsR2 knockdown attenuates CGX-mediated analgesia, supporting a critical role for NtsR2 in its effects. Mechanistically, CGX activation of NtsR2 inhibits Cav2.3 calcium channels, thereby reducing neuronal excitability and nociceptive signaling. Importantly, repeated CGX administration does not produce motor impairment or analgesic tolerance, further supporting the therapeutic potential of targeting spinal NtsR2 (Martin et al., 2022; Martin et al., 2025).

Several NtsR2-selective agonists modified at key residues have been developed with lengthened plasma half-lives and ability to treat pain without producing side effects, including CR-01–64, Compound 13, JMV5296, JMV431, and NT79 (Boules et al., 2009; Chartier et al., 2021; Eiselt et al., 2019a; Vivancos et al., 2021). Central JMV5296 administration in rats decreased pain in several pain models including formalin injection, CFA-induced mechanical hypersensitivity, and tail-flick test (Vivancos et al., 2021). Unlike Nts receptor pan-agonists and NtsR1 agonists, JMV5296 did not alter blood pressure or body temperature. JMV431, a similar Nts analog, produced antinociceptive effects in the tail-flick test in naïve rats, reduced formalin responding, and improved mechanical hypersensitivity and weight bearing following chronic constriction injury (CCI) (Sarret et al., 2005; Roussy et al., 2009; Tétreault et al., 2013). Another Nts analog modified to have increased plasma stability and selectivity to NtsR2 referred to as Compound 13 decreased pain scores in the formalin test when injected intrathecally and had no adverse effects in rats (Eiselt et al., 2019a). Another modified Nts agonist with preferential NtsR2 binding is CR-01–64 (Chartier et al., 2021). Intrathecal injection of CR-01–64 reduced CFA-induced mechanical hypersensitivity, thermal hypersensitivity, and spontaneous pain in the formalin test. CR-01–64 did not slow gut motility or cause hypothermia, although it did briefly cause hypotension that resolved within 2 minutes at a dose of 0.01 mg/kg injected intravenously and 15 min at a dose of 0.1 mg/kg. Like JMV5296 and Compound 13, CR-01–64 had to be injected intrathecally to have an analgesic effect, as systemic administration did not alter pain responding, suggesting these drugs are not blood-brain-barrier-permeable, and act on the central nervous system, limiting clinical potential. NT79 systemic administration showed antinociceptive effects decreasing acetic acid-induced writhing and no change to body temperature or blood pressure, suggesting that this compound is either acting on the DRG in the periphery or passes through the blood-brain barrier; the underlying mechanisms warrant further exploration. Curiously, NT79 did not have an antinociceptive effect on the hot plate test. ß-Lactotensin, a naturally-derived tetrapeptide and NtsR2 agonist, decreased mechanical hypersensitivity following CCI but did not improve weight bearing (Tétreault et al., 2013). Levocabastine, an antihistaminic drug that acts as a ligand for the low-affinity neurotensin receptor NtsR2, produced analgesia in the acetic acid writhing test, formalin test, and reduced mechanical hypersensitivity and increased weight bearing after CCI, but had no effect in the hot plate test (Dubuc et al., 1999b; Roussy et al., 2009; Sarret et al., 2005; Tétreault et al., 2013), suggesting an analgesic effect specifically in the context of injury-induced pain, without altering physiological thermal sensitivity. Levocabastine did not affect body temperature or neurotensin-induced hypothermia, indicating that NtsR2 contributes to visceral pain modulation but is unlikely to mediate the hypothermic effects of neurotensin (Dubuc et al., 1999b). Hence, NtsR2-selective agonists are attractive candidates for pain drug targets, but thus far they are limited by their lack of blood-brain barrier permeability.

## Limitations

Several important limitations currently constrain the translation of neurotensin-mediated antinociception to clinical pain treatment. First, most preclinical evidence for the antinociceptive effects of Nts is based on reflexive, stimulus-evoked behavioral assays, such as withdrawal responses to mechanical or thermal stimulation. These outcomes are fundamentally different from the self-reported pain experienced by patients, and a mechanism that reduces responsiveness to a noxious thermal stimulus does not necessarily translate into a meaningful reduction in clinical pain (Mogil, 2019; Sadler et al., 2022). Although a substantial body of preclinical work supports an antinociceptive role for Nts, direct clinical evidence remains extremely limited. This translational gap is partly explained by the adverse effects associated with currently available NtsR agonists (Dobbs and Morikawa, 2020; Tabarean, 2020), which have limited their clinical investigation, as well as the challenge of developing compounds capable of crossing the blood-brain barrier when central Nts signaling is the intended therapeutic target.

More recently, Guo et al. provided important translational evidence that pharmacological activation of NtsR signaling directly modulated human nervous tissue, demonstrating that SBI-810 reduced neuronal excitability in human DRG neurons and decreased NMDAR activity in human spinal cord slices (Guo et al., 2025). Moreover, alterations in endogenous neurotensin signaling have been reported in patients with chronic pain conditions, including irritable bowel syndrome and other visceral pain disorders, supporting the possibility that the Nts system participates in human pain pathophysiology (Didenko and Wood, 2025). However, studies directly measuring circulating or tissue Nts in well-characterized pain cohorts remain limited and are frequently based on relatively small sample sizes. For example, two studies examining Nts in fibromyalgia reported conflicting findings, with one identifying elevated Nts levels and another finding no significant difference (Navarro-Gonzalez et al., 2025; Tsilioni et al., 2016). This contrasts with metabolic disorders such as obesity, for which elevated circulating Nts has been more consistently reported (Gazit Shimoni et al., 2025; Li et al., 2016). Thus, larger clinical studies linking endogenous Nts levels with pain intensity, pain phenotype, and disease progression will be necessary to establish the clinical relevance of the Nts system.

Finally, an additional challenge is that peripheral and central Nts signaling can exert markedly different physiological effects. Peripheral Nts has been associated with increased appetite and weight gain, whereas central Nts signaling generally reduces food intake and body weight (Khan et al., 2024; Thompson and Leinninger, 2025). Although current preclinical studies largely support antinociceptive actions of Nts signaling both peripherally and centrally, differential or even opposing effects across these compartments in humans cannot be excluded. Together, these limitations emphasize the need for more clinically relevant pain outcomes in preclinical studies, greater characterization of the endogenous Nts system in patients, and the development of safe, receptor-selective NtsR modulators with appropriate peripheral or CNS exposure.

## Conclusion and future directions

In summary, accumulating evidence positions the Nts system as an important modulator of nociceptive processing across peripheral, spinal, and supraspinal levels of the nervous system. Nts signaling regulates nociceptor excitability, spinal neurotransmission, and pain-modulatory circuits involving the PAG, RVM, DRN, hypothalamus, and limbic structures, highlighting its broad influence on both sensory and affective dimensions of pain. Although pharmacological and genetic studies strongly support a role for Nts signaling in pain modulation, conflicting findings across experimental models underscore the complexity of this system and suggest that NtsR1- and NtsR2-mediated signaling exert distinct, context- and circuit-dependent effects. Importantly, the recent development of receptor-selective agonists and biased allosteric modulators has renewed interest in the therapeutic potential of targeting Nts receptors for analgesia. In particular, compounds such as SBI-810 demonstrate that selective modulation of NtsR1 signaling can produce antinociceptive effects while potentially avoiding some of the adverse effects associated with earlier Nts agonists.

Despite extensive preclinical evidence, the major limitation in the field remains the paucity of studies investigating the Nts system in humans. Available clinical studies have primarily examined circulating Nts concentrations in metabolic conditions or relatively small pain cohorts, limiting conclusions regarding the relationship between endogenous Nts signaling and clinical pain. Future studies should characterize Nts signaling in larger, well-phenotyped human pain cohorts and determine whether circulating or tissue Nts levels are associated with pain severity, chronicity, or specific pain phenotypes. Integration of these measurements with transcriptomic analyses of blood and tissues could identify molecular signatures associated with altered Nts signaling. Similarly, analysis of postmortem human nervous tissue could help define the cellular and anatomical distribution of Nts receptors and determine whether their expression is altered in chronic pain conditions.

Nevertheless, several observations provide an initial path toward clinical translation. Infusion of Nts has been investigated in healthy volunteers (NCT04179331), providing human safety and pharmacological information. Moreover, a Phase 1A safety study of the Nts analog CGX-1160 demonstrated that intrathecal administration was associated with analgesic effects in patients with neuropathic pain following spinal cord injury, with limited adverse effects (Sang et al., 2016). Although promising, this study included only four participants and therefore requires validation in larger clinical trials. Nts-related clinical interventions are also being investigated clinically outside the pain field, including studies related to appetite, metabolic regulation, and cancer (NCT04179331, NCT04186026, NCT00541762). Collectively, these studies can provide valuable information regarding the pharmacokinetics, safety, tolerability, and physiological consequences of manipulating the Nts system in humans that may facilitate future development of NtsR-targeting analgesics. Incorporating pain assessments into future clinical studies of Nts-targeting interventions, even when pain is not the primary outcome, could provide an efficient opportunity to identify potential analgesic signals in humans.

Future preclinical studies should also place greater emphasis on defining the receptor-, cell-, and circuit-specific functions of Nts signaling. Combining cell type-specific genetic manipulations with circuit mapping (Schroeder et al., 2019; Woodworth et al., 2017; Woodworth and Brown, 2018), electrophysiology, and transcriptomic approaches will be essential to determine how Nts differentially regulates nociceptive, affective, and descending modulatory circuits across acute and chronic pain states. Photopharmacological approaches using light-activated NtsR agonists or antagonists could be particularly valuable for achieving precise temporal and spatial control of receptor signaling, as demonstrated for other neuromodulatory systems, including opioid and noradrenergic signaling (Buczynski et al., 2025; McClain et al., 2023). Such approaches could help distinguish the effects of Nts signaling within individual brain regions and neuronal populations while also enabling mechanistic studies of NtsR pharmacology *ex vivo*.

Finally, Nts should not be considered solely as a pain-modulatory system. Nts is an important regulator of feeding, energy balance, stress responses, thermoregulation, and other physiological states. Consequently, obesity, metabolic disorders, psychiatric conditions, and other physiological or pathological states may modify Nts signaling and thereby influence pain sensitivity or chronification. Conversely, pharmacologically targeting NtsRs for pain could affect these other physiological functions. Understanding these interactions will therefore be essential both for predicting potential adverse effects and for identifying patient populations that may particularly benefit from NtsR-targeted therapies. Together, advances in receptor-selective pharmacology, circuit-specific approaches, and human translational studies may ultimately determine whether the strong preclinical evidence supporting Nts-mediated antinociception can be translated into a new class of non-opioid analgesics.

## References

[B1] Aaron R. V. Ravyts S. G. Carnahan N. D. Bhattiprolu K. Harte N. McCaulley C. C. (2025). Prevalence of depression and anxiety among adults with chronic pain: a systematic review and meta-analysis. JAMA Network Open 8 (3), e250268. 10.1001/jamanetworkopen.2025.0268 PMC11889470 40053352

[B2] Abdul M. Yan H. Q. Zhao W. N. Lyu X. B. Xu Z. Yu X. L. (2022). VTA-NAc glutaminergic projection involves in the regulation of pain and pain-related anxiety. Front. Mol. Neurosci. 15, 1083671. 10.3389/fnmol.2022.1083671 PMC9798784 36590915

[B3] Ables J. L. Park K. Ibañez-Tallon I. (2023). Understanding the habenula: a major node in circuits regulating emotion and motivation. Pharmacol. Res. 190, 106734. 10.1016/j.phrs.2023.106734 PMC11081310 36933754

[B4] Adams J. E. (1976). Naloxone reversal of analgesia produced by brain stimulation in the human. Pain 2 (2), 161–166. 10.1016/0304-3959(76)90111-1 800249

[B6] Aimone L. D. Bauer C. A. Gebhart G. F. (1988). Brain-stem relays mediating stimulation-produced antinociception from the lateral hypothalamus in the rat. J. Neurosci. 8 (7), 2652–2663. 10.1523/JNEUROSCI.08-07-02652.1988 3249250 PMC6569510

[B7] Akil H. Mayer D. J. Liebeskind J. C. (1976). Antagonism of stimulation-produced analgesia by naloxone, a narcotic antagonist. Science 191 (4230), 961–962. 10.1126/science.1251210 1251210

[B8] Al-Rodhan N. R. Richelson E. Gilbert J. A. McCormick D. J. Kanba K. S. Pfenning M. A. (1991). Structure-antinociceptive activity of neurotensin and some novel analogues in the periaqueductal gray region of the brainstem. Brain Res. 557 (1–2), 227–235. 10.1016/0006-8993(91)90139-m 1660754

[B9] Alexander M. J. Leeman S. E. (1994). Estrogen-inducible neurotensin immunoreactivity in the preoptic area of the female rat. J. Comp. Neurology 345 (4), 496–509. 10.1002/cne.903450403 7962697

[B10] Alexander M. J. Leeman S. E. (1998). Widespread expression in adult rat forebrain of mRNA encoding high-affinity neurotensin receptor. J. Comp. Neurology 402 (4), 475–500. 10.1002/(SICI)1096-9861(19981228)402:4<475::AID-CNE4>3.0.CO;2-9 9862322

[B11] Ashina M. (2020). Migraine. N. Engl. J. Med. 383 (19), 1866–1876. 10.1056/NEJMra1915327 33211930

[B13] Asselin M. L. Dubuc I. Coquerel A. Costentin J. (2001). Localization of neurotensin NTS2 receptors in rat brain, using. Neuroreport 12 (5), 1087–1091. 10.1097/00001756-200104170-00044 11303751

[B14] Bagley E. E. Ingram S. L. (2020). Endogenous opioid peptides in the descending pain modulatory circuit. Neuropharmacology 173, 108131. 10.1016/j.neuropharm.2020.108131 32422213 PMC7313723

[B15] Barbaro N. M. (1988). Studies of PAG/PVG stimulation for pain relief in humans. Prog. Brain Res. 77, 165–173. 10.1016/s0079-6123(08)62783-1 2464178

[B16] Barik A. Thompson J. H. Seltzer M. Ghitani N. Chesler A. T. (2018). A brainstem-spinal circuit controlling nocifensive behavior. Neuron 100 (6), 1491–1503.e3. 10.1016/j.neuron.2018.10.037 30449655

[B17] Basbaum A. I. Bautista D. M. Scherrer G. Julius D. (2009). Cellular and molecular mechanisms of pain. Cell 139 (2), 267–284. 10.1016/j.cell.2009.09.028 19837031 PMC2852643

[B18] Behbehani M. M. Pert A. (1984). A mechanism for the analgesic effect of neurotensin as revealed by behavioral and electrophysiological techniques. Brain Res. 324 (1), 35–42. 10.1016/0006-8993(84)90619-x 6518391

[B19] Behbehani M. M. Park M. R. Clement M. E. (1988). Interactions between the lateral hypothalamus and the periaqueductal gray. J. Neurosci. 8 (8), 2780–2787. 10.1523/JNEUROSCI.08-08-02780.1988 2900881 PMC6569401

[B20] Beier K. T. Steinberg E. E. DeLoach K. E. Xie S. Miyamichi K. Schwarz L. (2015). Circuit architecture of VTA dopamine neurons revealed by systematic input-output mapping. Cell 162 (3), 622–634. 10.1016/j.cell.2015.07.015 26232228 PMC4522312

[B21] Bernard J. F. Bester H. Besson J. M. (1996). “Chapter 14 involvement of the spino-parabrachio -amygdaloid and -hypothalamic pathways in the autonomic and affective emotional aspects of pain,” in The Emotional Motor System. Elsevier, pp. 243–255. 10.1016/S0079-6123(08)61868-3 8782523

[B23] Bhuiyan S. A. Xu M. Yang L. Semizoglou E. Bhatia P. Pantaleo K. I. (2024). Harmonized cross-species cell atlases of trigeminal and dorsal root ganglia. Sci. Adv. 10 (25), eadj9173. 10.1126/sciadv.adj9173 PMC11804847 38905344

[B24] Bissette G. Nemeroff C. B. Loosen P. T. Prange A. J. Lipton M. A. (1976). Hypothermia and intolerance to cold induced by intracisternal administration of the hypothalamic peptide neurotensin. Nature 262 (5569), 607–609. 10.1038/262607a0 8728

[B25] Boules M. Shaw A. Liang Y. Barbut D. Richelson E. (2009). NT69L, a novel analgesic, shows synergy with morphine. Brain Res. 1294, 22–28. 10.1016/j.brainres.2009.07.086 19651107

[B26] Boules M. Li Z. Smith K. Fredrickson P. Richelson E. (2013). Diverse roles of neurotensin agonists in the central nervous system. Front. Endocrinology 4, 36. 10.3389/fendo.2013.00036 23526754 PMC3605594

[B27] Bradman M. J. G. Ferrini F. Salio C. Merighi A. (2015). Practical mechanical threshold estimation in rodents using von Frey hairs/Semmes-Weinstein monofilaments: towards a rational method. J. Neurosci. Methods 255, 92–103. 10.1016/j.jneumeth.2015.08.010 26296284

[B28] Braz J. M. Nassar M. A. Wood J. N. Basbaum A. I. (2005). Parallel ‘pain’ pathways arise from subpopulations of primary afferent nociceptor. Neuron 47 (6), 787–793. 10.1016/j.neuron.2005.08.015 16157274

[B29] Bredeloux P. Costentin J. Dubuc I. (2006). Interactions between NTS2 neurotensin and opioid receptors on two nociceptive responses assessed on the hot plate test in mice. Behav. Brain Res. 175 (2), 399–407. 10.1016/j.bbr.2006.09.016 17074405

[B31] Bridges D. Thompson S. W. Rice A. S. (2001). Mechanisms of neuropathic pain. Br. J. Anaesth. 87 (1), 12–26. 10.1093/bja/87.1.12 11460801

[B32] Brown J. A. Wright A. Bugescu R. Christensen L. Olson D. P. Leinninger G. M. (2019). Distinct subsets of lateral hypothalamic neurotensin neurons are activated by leptin or dehydration. Sci. Rep. 9 (1), 1873. 10.1038/s41598-018-38143-9 30755658 PMC6372669

[B33] Buchanan W. W. Rainsford K. D. Kean C. A. Kean W. F. (2024). Narcotic analgesics. Inflammopharmacology 32 (1), 23–28. 10.1007/s10787-023-01304-y 37515654

[B34] Bucklan J. Ahmed Z. (2020). CGRP antagonists for decreasing migraine frequency: new options, long overdue. Clevel. Clin. Journal Medicine 87 (4), 211–218. 10.3949/ccjm.87a.19048 32238376

[B35] Buczynski S. A. Li A. Chang-Weinberg J. Nawsheen S. S. Banghart M. R. (2025). A chemically stable photocaged noradrenaline. ACS Chem. Neurosci. 16 (15), 2935–2944. 10.1021/acschemneuro.5c00241 PMC12333006 40626729

[B36] Buhler A. V. Choi J. Proudfit H. K. Gebhart G. F. (2005). Neurotensin activation of the NTR1 on spinally-projecting serotonergic neurons in the rostral ventromedial medulla is antinociceptive. Pain 114 (1–2), 285–294. 10.1016/j.pain.2004.12.031 15733655

[B38] Bushnell M. C. Ceko M. Low L. A. (2013). Cognitive and emotional control of pain and its disruption in chronic pain. Nat. Rev. Neurosci. 14 (7), 502–511. 10.1038/nrn3516 23719569 PMC4465351

[B39] Busse J. W. Wang L. Kamaleldin M. Craigie S. Riva J. J. Montoya L. (2018). Opioids for chronic noncancer pain: a systematic review and meta-analysis. J. Am. Med. Assoc. 320 (23), 2448–2460. 10.1001/jama.2018.18472 30561481 PMC6583638

[B40] Carraway R. Leeman S. E. (1973). The isolation of a new hypotensive peptide, neurotensin, from bovine hypothalami. J. Biol. Chem. 248 (19), 6854–6861. 4745447

[B41] Carraway R. Leeman S. E. (1975). The amino acid sequence of a hypothalamic peptide, neurotensin. J. Biol. Chem. 250 (5), 1907–1911. 1167549

[B42] Carraway R. Cochrane D. E. Lansman J. B. Leeman S. E. Paterson B. M. Welch H. J. (1982). Neurotensin stimulates exocytotic histamine secretion from rat mast cells and elevates plasma histamine levels. J. Physiology 323, 403–414. 10.1113/jphysiol.1982.sp014080 6178820 PMC1250364

[B43] Casarrubea M. Faulisi F. Magnusson M. S. Crescimanno G. (2016). The effects of morphine on the temporal structure of wistar rat behavioral response to pain in hot-plate. Psychopharmacology 233 (15–16), 2891–2900. 10.1007/s00213-016-4334-6 27235015

[B44] Chalon P. Vita N. Kaghad M. Guillemot M. Bonnin J. Delpech B. (1996). Molecular cloning of a levocabastine-sensitive neurotensin binding site. FEBS Lett. 386 (2–3), 91–94. 10.1016/0014-5793(96)00397-3 8647296

[B45] Chantalat E. Valera M. C. Vaysse C. Noirrit E. Rusidze M. Weyl A. (2020). Estrogen receptors and endometriosis. Int. J. Mol. Sci. 21 (8), 2815. 10.3390/ijms21082815 32316608 PMC7215544

[B46] Chaplan S. R. Bach F. W. Pogrel J. W. Chung J. M. Yaksh T. L. (1994). Quantitative assessment of tactile allodynia in the rat paw. J. Neurosci. Methods 53 (1), 55–63. 10.1016/0165-0270(94)90144-9 7990513

[B47] Chartier M. Desgagné M. Sousbie M. Rumsby C. Chevillard L. Théroux L. (2021). Pharmacodynamic and pharmacokinetic profiles of a neurotensin receptor type 2 (NTS2) analgesic macrocyclic analog. Biomed. and Pharmacother. 141, 111861. 10.1016/j.biopha.2021.111861 34229249

[B48] Chen Y. Song Y. Wang H. Zhang Y. Hu X. Wang K. (2022). Distinct neural networks derived from galanin-containing nociceptors and neurotensin-expressing pruriceptors. Proc. Natl. Acad. Sci. U. S. A. 119 (33), e2118501119. 10.1073/pnas.2118501119 PMC9388111 35943985

[B49] Clineschmidt B. V. McGuffin J. C. (1977). Neurotensin administered intracisternally inhibits responsiveness of mice to noxious stimuli. Eur. J. Pharmacol. 46 (4), 395–396. 10.1016/0014-2999(77)90236-9 201475

[B50] Cohen F. Brooks C. V. Sun D. Buse D. C. Reed M. L. Fanning K. M. (2024). Prevalence and burden of migraine in the United States: a systematic review. Headache 64 (5), 516–532. 10.1111/head.14709 38700185

[B51] Conti P. D'Ovidio C. Conti C. Gallenga C. E. Lauritano D. Caraffa A. (2019). Progression in migraine: role of mast cells and pro-inflammatory and anti-inflammatory cytokines. Eur. J. Pharmacol. 844, 87–94. 10.1016/j.ejphar.2018.12.004 30529470

[B52] Cooke J. H. Patterson M. Patel S. R. Smith K. L. Ghatei M. A. Bloom S. R. (2009). Peripheral and central administration of xenin and neurotensin suppress food intake in rodents. Obesity 17 (6), 1135–1143. 10.1038/oby.2008.652 19214175

[B53] Corder G. Castro D. C. Bruchas M. R. Scherrer G. (2018). Endogenous and exogenous opioids in pain. Annu. Rev. Neurosci. 41, 453–473. 10.1146/annurev-neuro-080317-061522 29852083 PMC6428583

[B54] Cowie A. M. Stucky C. L. (2019). A mouse model of postoperative pain. Bio-protocol 9 (2), e3140. 10.21769/BioProtoc.3140 30820443 PMC6390978

[B55] Dafny N. Dong W. Q. Prieto-Gomez C. Reyes-Vazquez C. Stanford J. Qiao J. T. (1996). Lateral hypothalamus: site involved in pain modulation. Neuroscience 70 (2), 449–460. 10.1016/0306-4522(95)00358-4 8848153

[B56] Davis G. O. Ma S. Kernan M. Sumarli D. Loveless M. C. Schroeder K. W. (2026). Periaqueductal gray neurotensin neurons drive simultaneous threat response and reinforcement. BioRxiv. 10.64898/2026.02.16.706198

[B57] De Preter C. C. Heinricher M. M. (2024). The ‘in’s and out’s’ of descending pain modulation from the rostral ventromedial medulla. Trends Neurosci. 47 (6), 447–460. 10.1016/j.tins.2024.04.006 PMC11168876 38749825

[B58] de Souza S. Hua H. Laumet S. Sim J. Vanacker C. Bavencoffe A. (2026). C-C motif chemokine ligand 12 (CCL12) is a critical chemokine driving postoperative pain. Pain 167 (5), 1098–1109. 10.1097/j.pain.0000000000003936 PMC13213411 41800753

[B59] Decosterd I. Woolf C. J. (2000). Spared nerve injury: an animal model of persistent peripheral neuropathic pain. Pain 87 (2), 149–158. 10.1016/S0304-3959(00)00276-1 10924808

[B60] Demeule M. Beaudet N. Régina A. Besserer-Offroy É. Murza A. Tétreault P. (2014). Conjugation of a brain-penetrant peptide with neurotensin provides antinociceptive properties. J. Clin. Investigation 124 (3), 1199–1213. 10.1172/JCI70647 24531547 PMC3934173

[B61] Deuis J. R. Dvorakova L. S. Vetter I. (2017). Methods used to evaluate pain behaviors in rodents. Front. Mol. Neurosci. 10, 284. 10.3389/fnmol.2017.00284 28932184 PMC5592204

[B62] Deyama S. Nakagawa T. Kaneko S. Uehara T. Minami M. (2007). Involvement of the bed nucleus of the stria terminalis in the negative affective component of visceral and somatic pain in rats. Behav. Brain Res. 176 (2), 367–371. 10.1016/j.bbr.2006.10.021 17101179

[B64] Didenko O. Wood J. N. (2025). Arrestins and opioid-independent analgesia. Cell 188 (16), 4175–4177. 10.1016/j.cell.2025.07.008 40780184

[B66] Dobbs L. K. Morikawa H. (2020). Biasing neurotensin receptor signaling to arrest psychostimulant abuse. Cell 181 (6), 1205–1206. 10.1016/j.cell.2020.05.009 32470394

[B68] Dowell D. Ragan K. R. Jones C. M. Baldwin G. T. Chou R. (2022). CDC clinical practice guideline for prescribing opioids for pain - United States, 2022. MMWR. Recomm. Rep. Morb. Mortal. Wkly. Rep. 71 (3), 1–95. 10.15585/mmwr.rr7103a1 PMC9639433 36327391

[B69] Drake R. A. Steel K. A. Apps R. Lumb B. M. Pickering A. E. (2021). Loss of cortical control over the descending pain modulatory system determines the development of the neuropathic pain state in rats. eLife 10, e65156. 10.7554/eLife.65156 PMC7895525 33555256

[B71] Dubuc I. Sarret P. Labbé-Jullié C. Botto J. M. Honoré E. Bourdel E. (1999a). Identification of the receptor subtype involved in the analgesic effect of neurotensin. J. Neurosci. 19 (1), 503–510. 10.1523/JNEUROSCI.19-01-00503.1999 9870978 PMC6782393

[B72] Dubuc I. Remande S. Costentin J. (1999b). The partial agonist properties of levocabastine in neurotensin-induced analgesia. Eur. J. Pharmacol. 381 (1), 9–12. 10.1016/S0014-2999(99)00554-3 10528128

[B73] Edvinsson L. Haanes K. A. Warfvinge K. Krause D. N. (2018). CGRP as the target of new migraine therapies - successful translation from bench to clinic. Nat. Rev. Neurol. 14 (6), 338–350. 10.1038/s41582-018-0003-1 29691490

[B74] Eiselt E. Côté J. Longpré J.-M. Blais V. Sarret P. Gendron L. (2019a). The combination of opioid and neurotensin receptor agonists improves their analgesic/adverse effect ratio. Eur. J. Pharmacol. 848, 80–87. 10.1016/j.ejphar.2019.01.048 30707956

[B75] Eiselt E. Gonzalez S. Martin C. Chartier M. Betti C. Longpré J.-M. (2019b). Neurotensin analogues containing cyclic surrogates of tyrosine at position 11 improve NTS2 selectivity leading to analgesia without hypotension and hypothermia. ACS Chem. Neurosci. 10 (11), 4535–4544. 10.1021/acschemneuro.9b00390 31589400

[B76] Engle M. P. Merrill M. A. Marquez De Prado B. Hammond D. L. (2012). Spinal nerve ligation decreases γ-aminobutyric acidB receptors on specific populations of immunohistochemically identified neurons in L5 dorsal root ganglion of the rat. J. Comp. Neurology 520 (8), 1663–1677. 10.1002/cne.23005 22120979 PMC3902039

[B77] Espejo E. F. Mir D. (1993). Structure of the rat’s behaviour in the hot plate test. Behav. Brain Res. 56 (2), 171–176. 10.1016/0166-4328(93)90035-o 8240711

[B78] Faget L. Osakada F. Duan J. Ressler R. Johnson A. B. Proudfoot J. A. (2016). Afferent inputs to neurotransmitter-defined cell types in the ventral tegmental area. Cell Reports 15 (12), 2796–2808. 10.1016/j.celrep.2016.05.057 PMC4917450 27292633

[B79] Fantegrossi W. E. Ko M. C. H. Woods J. H. Richelson E. (2005). Antinociceptive, hypothermic, hypotensive, and reinforcing effects of a novel neurotensin receptor agonist, NT69L, in rhesus monkeys. Pharmacol. Biochem. Behav. 80 (2), 341–349. 10.1016/j.pbb.2004.12.005 15680187

[B80] Faull R. L. Villiger J. W. Dragunow M. (1989). Neurotensin receptors in the human spinal cord: a quantitative autoradiographic study. Neuroscience 29 (3), 603–613. 10.1016/0306-4522(89)90134-6 2544824

[B81] Feifel D. Goldenberg J. Melendez G. Shilling P. D. (2010). The acute and subchronic effects of a brain-penetrating, neurotensin-1 receptor agonist on feeding, body weight and temperature. Neuropharmacology 58 (1), 195–198. 10.1016/j.neuropharm.2009.07.001 19596358 PMC2784133

[B82] Feldberg R. S. Cochrane D. E. Carraway R. E. Brown E. Sawyer R. Hartunian M. (1998). Evidence for a neurotensin receptor in rat serosal mast cells. Inflamm. Res. 47 (6), 245–250. 10.1007/s000110050325 9683031

[B83] Ferraro L. Tomasini M. C. Mazza R. Fuxe K. Fournier J. Tanganelli S. (2008). Neurotensin receptors as modulators of glutamatergic transmission. Brain Res. Rev. 58 (2), 365–373. 10.1016/j.brainresrev.2007.11.001 18096238

[B84] Fields H. L. Heinricher M. M. (1985). Anatomy and physiology of a nociceptive modulatory system. Philosophical Trans. R. Soc. Lond. Ser. B, Biol. Sci. 308 (1136), 361–374. 10.1098/rstb.1985.0037 2858889

[B85] Fields H. L. Heinricher M. M. Mason P. (1991). Neurotransmitters in nociceptive modulatory circuits. Annu. Rev. Neurosci. 14, 219–245. 10.1146/annurev.ne.14.030191.001251 1674413

[B86] Finnerup N. B. Attal N. Haroutounian S. McNicol E. Baron R. Dworkin R. H. (2015). Pharmacotherapy for neuropathic pain in adults: a systematic review and meta-analysis. Lancet Neurol. 14 (2), 162–173. 10.1016/S1474-4422(14)70251-0 25575710 PMC4493167

[B87] Flores-García M. Rizzo A. Garçon-Poca M. Z. Fernández-Dueñas V. Bonaventura J. (2023). Converging circuits between pain and depression: the ventral tegmental area as a therapeutic hub. Front. Pharmacology 14, 1278023. 10.3389/fphar.2023.1278023 PMC10577189 37849731

[B88] Friebel U. Eickhoff S. B. Lotze M. (2011). Coordinate-based meta-analysis of experimentally induced and chronic persistent neuropathic pain. Neuroimage 58 (4), 1070–1080. 10.1016/j.neuroimage.2011.07.022 21798355 PMC8018239

[B89] Frigerio B. Ravazola M. Ito S. Buffa R. Capella C. Solcia E. (1977). Histochemical and ultrastructural identification of neurotensin cells in the dog ileum. Histochemistry 54 (2), 123–131. 10.1007/BF00489670 72747

[B90] Fu W. Lai Y. Li K. Yang Y. Guo X. Gong Q. (2025). Neurotensin-neurotensin receptor 2 signaling in adipocytes suppresses food intake through regulating ceramide metabolism. Cell Res. 35 (2), 117–131. 10.1038/s41422-024-01038-8 PMC11770130 39748047

[B91] Gaffney C. M. Muwanga G. Shen H. Tawfik V. L. Shepherd A. J. (2022). Mechanical conflict-avoidance assay to measure pain behavior in mice. J. of Vis. Exp. Prepr. 180, e63454. 10.3791/63454 PMC9058981 35253785

[B92] Garelja M. L. Hay D. L. (2022). A narrative review of the calcitonin peptide family and associated receptors as migraine targets: calcitonin gene-related peptide and beyond. Headache 62 (9), 1093–1104. 10.1111/head.14388 PMC9613588 36226379

[B93] Gazit Shimoni N. Tose A. J. Seng C. Jin Y. Lukacsovich T. Yang H. (2025). Changes in neurotensin signalling drive Hedonic devaluation in obesity. Nature 641 (8065), 1238–1247. 10.1038/s41586-025-08748-y PMC12119351 40140571

[B94] Gilliam-Vigh H. Jorsal T. Nielsen S. W. Forman J. L. Pedersen J. Poulsen S. S. (2023). Expression of neurotensin and its receptors along the intestinal tract in type 2 diabetes patients and healthy controls. J. Clin. Endocrinol. Metabolism 108 (9), 2211–2216. 10.1210/clinem/dgad146 36916883

[B96] Golinska M. Wołyniak M. Kulesza P. Fendler W. (2025). Neuroinflammation is responsible for pain in endometriosis - targeting the JAK-STAT pathway and mast cell activation. Front. Immunol. 16, 1621178. 10.3389/fimmu.2025.1621178 PMC12425711 40948761

[B97] Gomez K. Santiago U. Nelson T. S. Allen H. N. Calderon-Rivera A. Hestehave S. (2023). A peptidomimetic modulator of the CaV2.2 N-type calcium channel for chronic pain. Proc. Natl. Acad. Sci. U. S. A. 120 (47), e2305215120. 10.1073/pnas.2305215120 PMC10666126 37972067

[B98] Gray T. S. Magnuson D. J. (1992). Peptide immunoreactive neurons in the amygdala and the bed nucleus of the stria terminalis project to the midbrain central gray in the rat. Peptides 13 (3), 451–460. 10.1016/0196-9781(92)90074-D 1381826

[B99] Gregory N. S. Harris A. L. Robinson C. R. Dougherty P. M. Fuchs P. N. Sluka K. A. (2013). An overview of animal models of pain: disease models and outcome measures. J. Pain 14 (11), 1255–1269. 10.1016/j.jpain.2013.06.008 24035349 PMC3818391

[B100] Gui X. Carraway R. E. Dobner P. R. (2004). Endogenous neurotensin facilitates visceral nociception and is required for stress-induced antinociception in mice and rats. Neuroscience 126 (4), 1023–1032. 10.1016/j.neuroscience.2004.04.034 15207335

[B101] Guo R. Chen O. Zhou Y. Bang S. Chandra S. Li Y. (2025). Arrestin-biased allosteric modulator of neurotensin receptor 1 alleviates acute and chronic pain. Cell 188 (16), 4332–4349. 10.1016/j.cell.2025.04.038 PMC12335375 40393456

[B102] Gürkan F. Ece A. Haspolat K. Dikici B. (2000). Parental history of migraine and bronchial asthma in children. Allergologia Immunopathol. 28 (1), 15–17. 10757853

[B103] Guy G. P. Miller G. F. Legha J. K. Rikard S. M. Strahan A. E. Mikosz C. (2025). Economic costs of chronic pain-united states, 2021. Med. Care 63 (9), 679–685. 10.1097/MLR.0000000000002181 PMC13284907 40730349

[B104] Han S. Soleiman M. T. Soden M. E. Zweifel L. S. Palmiter R. D. (2015). Elucidating an affective pain circuit that creates a threat memory. Cell 162 (2), 363–374. 10.1016/j.cell.2015.05.057 PMC4512641 26186190

[B105] Hara Y. Shiosaka S. Senba E. Sakanaka M. Inagaki S. Takagi H. (1982). Ontogeny of the neurotensin-containing neuron system of the rat: immunohistochemical analysis. I. Forebrain and diencephalon. J. Comp. Neurology 208 (2), 177–195. 10.1002/cne.902080207 7119156

[B106] Hargreaves K. Dubner R. Brown F. Flores C. Joris J. (1988). A new and sensitive method for measuring thermal nociception in cutaneous hyperalgesia. Pain 32 (1), 77–88. 10.1016/0304-3959(88)90026-7 3340425

[B108] Hawkins M. F. Baker J. D. Baumeister A. A. (1989). Neurotensin-induced polydipsia: a structure-activity study. Brain Res. 487 (1), 188–191. 10.1016/0006-8993(89)90957-8 2752285

[B110] Heinricher M. M. (2016). Pain modulation and the transition from acute to chronic pain. Adv. Exp. Med. Biol. 904, 105–115. 10.1007/978-94-017-7537-3_8 26900066

[B111] Heinricher M. M. Neubert M. J. (2004). Neural basis for the hyperalgesic action of cholecystokinin in the rostral ventromedial medulla. J. Neurophysiology 92 (4), 1982–1989. 10.1152/jn.00411.2004 15152023

[B112] Heinricher M. M. Morgan M. M. Fields H. L. (1992). Direct and indirect actions of morphine on medullary neurons that modulate nociception. Neuroscience 48 (3), 533–543. 10.1016/0306-4522(92)90400-v 1603332

[B113] Heinricher M. M. Tavares I. Leith J. L. Lumb B. M. (2009). Descending control of nociception: specificity, recruitment and plasticity. Brain Res. Rev. 60 (1), 214–225. 10.1016/j.brainresrev.2008.12.009 19146877 PMC2894733

[B114] Honkaniemi J. Pelto-Huikko M. Isola J. Rechardt L. (1990). Simultaneous localization of calcitonin gene-related peptide and neurotensin in rat central amygdaloid nucleus. Neurosci. Lett. 113 (1), 1–6. 10.1016/0304-3940(90)90484-q 2366947

[B115] Hu S. Zhang Z. Xie W. Mu M. (2026). State-dependent neurotensin modulation in the brain: from network dynamics to pharmacological opportunity. Curr. Opin. Pharmacol. 89, 102633. 10.1016/j.coph.2026.102633 42176564

[B116] Huang K. W. Ochandarena N. E. Philson A. C. Hyun M. Birnbaum J. E. Cicconet M. (2019). Molecular and anatomical organization of the dorsal raphe nucleus. eLife 8, e46464. 10.7554/eLife.46464 31411560 PMC6726424

[B117] Hughes F. M. Shaner B. E. May L. A. Zotian L. Brower J. O. Woods R. J. (2010). Identification and functional characterization of a stable, centrally active derivative of the neurotensin (8-13) fragment as a potential first-in-class analgesic. J. Med. Chem. 53 (12), 4623–4632. 10.1021/jm100092s 20481538 PMC2908295

[B118] Hung C.-H. Wang J.C.-F. Strichartz G. R. (2015). Spontaneous chronic pain after experimental thoracotomy revealed by conditioned place preference: morphine differentiates tactile evoked pain from spontaneous pain. J. Pain 16 (9), 903–912. 10.1016/j.jpain.2015.06.006 26116369 PMC4556597

[B119] Ibuki T. Hama A. T. Wang X. T. Pappas G. D. Sagen J. (1997). Loss of GABA-Immunoreactivity in the spinal dorsal horn of rats with peripheral nerve injury and promotion of recovery by adrenal medullary grafts. Neuroscience 76 (3), 845–858. 10.1016/s0306-4522(96)00341-7 9135056

[B120] Inyang K. E. Sim J. Clark K. B. Geron M. Monahan K. Evans C. (2025). Upregulation of delta opioid receptor by meningeal interleukin-10 prevents relapsing pain. Brain, Behav. Immun. 123, 399–410. 10.1016/j.bbi.2024.09.031 PMC11624093 39349285

[B122] Jacobsen L. Madsen P. Jacobsen C. Nielsen M. S. Gliemann J. Petersen C. M. (2001). Activation and functional characterization of the mosaic receptor SorLA/LR11. J. Biol. Chem. 276 (25), 22788–22796. 10.1074/jbc.M100857200 11294867

[B123] Ji R.-R. Chamessian A. Zhang Y.-Q. (2016). Pain regulation by non-neuronal cells and inflammation. Science 354 (6312), 572–577. 10.1126/science.aaf8924 27811267 PMC5488328

[B124] Kalivas P. W. Jennes L. Nemeroff C. B. Prange A. J. (1982). Neurotensin: topographical distribution of brain sites involved in hypothermia and antinociception. J. Comp. Neurology 210 (3), 225–238. 10.1002/cne.902100303 7142439

[B125] Kamel R. Leroy J. Vandecasteele G. Fischmeister R. (2023). Cyclic nucleotide phosphodiesterases as therapeutic targets in cardiac hypertrophy and heart failure. Nat. Rev. Cardiol. 20 (2), 90–108. 10.1038/s41569-022-00756-z 36050457

[B126] Kawarada S. Unno T. Ohashi H. Komori S. (2000). Neurotensin-induced Cl(-) current in guinea-pig dorsal root ganglion cells. Eur. J. Pharmacol. 404 (1–2), 69–78. 10.1016/s0014-2999(00)00604-x 10980264

[B127] Khan R. Lee B. Inyang K. Bemis H. Bugescu R. Laumet G. (2024). Neurotensin-expressing lateral hypothalamic neurons alleviate neuropathic and inflammatory pain *via* neurotensin receptor signaling. Neurobiol. Pain (Elsevier) 16, 100172. 10.1016/j.ynpai.2024.100172 PMC11550133 39524478

[B128] Khan R. Laumet G. Leinninger G. M. (2024). Hungry for relief: potential for neurotensin to address comorbid obesity and pain. Appetite 200, 107540. 10.1016/j.appet.2024.107540 38852785

[B129] Kiritoshi T. Yakhnitsa V. Singh S. Wilson T. D. Chaudhry S. Neugebauer B. (2024). Cells and circuits for amygdala neuroplasticity in the transition to chronic pain. Cell Reports 43 (9), 114669. 10.1016/j.celrep.2024.114669 PMC11473139 39178115

[B130] Kitabgi P. (1989). Neurotensin modulates dopamine neurotransmission at several levels along brain dopaminergic pathways. Neurochem. Int. 14 (2), 111–119. 10.1016/0197-0186(89)90110-1 20504406

[B131] Kitabgi P. (2006). Inactivation of neurotensin and neuromedin N by Zn metallopeptidases. Peptides 27 (10), 2515–2522. 10.1016/j.peptides.2005.12.017 16904239

[B132] Konnova E. A. Deftu A. F. Chu Sin Chung P. Pertin M. Kirschmann G. Decosterd I. (2023). Characterisation of GFAP-Expressing glial cells in the dorsal root ganglion after spared nerve injury. Int. J. Mol. Sci. 24 (21), 15559. 10.3390/ijms242115559 PMC10647921 37958541

[B133] KuKanich B. Lascelles B. D. X. Papich M. G. (2005). Use of a von Frey device for evaluation of pharmacokinetics and pharmacodynamics of morphine after intravenous administration as an infusion or multiple doses in dogs. Am. J. Veterinary Res. 66 (11), 1968–1974. 10.2460/ajvr.2005.66.1968 16334958

[B134] Kuroyanagi H. Kusakabe H. Ikegaya Y. Matsumoto N. (2026). Acetic acid-induced writhing is temporally dissociated from gastrointestinal motility in freely moving rats. Biol. and Pharm. Bull. 49 (5), 818–821. 10.1248/bpb.b26-00009 42144371

[B135] Kurt G. Kodur N. Quiles C. R. Reynolds C. Eagle A. Mayer T. (2022). Time to drink: activating lateral hypothalamic area neurotensin neurons promotes intake of fluid over food in a time-dependent manner. Physiology and Behav. 247, 113707. 10.1016/j.physbeh.2022.113707 PMC8844224 35063424

[B136] Kyriatzis G. Bernard A. Bôle A. Pflieger G. Chalas P. Masse M. (2021). Neurotensin receptor 2 is induced in astrocytes and brain endothelial cells in relation to neuroinflammation following pilocarpine-induced seizures in rats. Glia 69 (11), 2618–2643. 10.1002/glia.24062 34310753

[B137] Lackovic J. Price T. J. Dussor G. (2021). *De novo* protein synthesis is necessary for priming in preclinical models of migraine. Cephalalgia 41 (2), 237–246. 10.1177/0333102420970514 33200943

[B138] Laffray S. Bouali-Benazzouz R. Papon M. A. Favereaux A. Jiang Y. Holm T. (2012). Impairment of GABAB receptor dimer by endogenous 14-3-3ζ in chronic pain conditions. EMBO J. 31 (15), 3239–3251. 10.1038/emboj.2012.161 22692127 PMC3411072

[B139] Lafrance M. Roussy G. Belleville K. Maeno H. Beaudet N. Wada K. (2010). Involvement of NTS2 receptors in stress-induced analgesia. Neuroscience 166 (2), 639–652. 10.1016/j.neuroscience.2009.12.042 20035838

[B140] Langford D. J. Bailey A. L. Chanda M. L. Clarke S. E. Drummond T. E. Echols S. (2010). Coding of facial expressions of pain in the laboratory mouse. Nat. Methods 7 (6), 447–449. 10.1038/nmeth.1455 20453868

[B141] Laumet G. (2026). Closing in on the causes of chronic neuropathic pain. eLife 15, e111218. 10.7554/eLife.111218 PMC12999170 41849200

[B142] Laumet G. Garriga J. Chen S. R. Zhang Y. Li D. P. Smith T. M. (2015). G9a is essential for epigenetic silencing of K(+) channel genes in acute-to-chronic pain transition. Nat. Neurosci. 18 (12), 1746–1755. 10.1038/nn.4165 26551542 PMC4661086

[B143] Laumet G. Bavencoffe A. Edralin J. D. Huo X. J. Walters E. T. Dantzer R. (2020). Interleukin-10 resolves pain hypersensitivity induced by cisplatin by reversing sensory neuron hyperexcitability. Pain 161 (10), 2344–2352. 10.1097/j.pain.0000000000001921 32427749 PMC7962468

[B144] Laumet S. Edwards A. M. Inyang K. E. Laumet G. (2025). Unexpected role of tnfα signaling in the resolution of postoperative pain in mice. J. Pain Research 18, 6057–6063. 10.2147/JPR.S543971 PMC12620509 41257213

[B145] Lavigne G. J. Hargreaves K. M. Schmidt E. A. Dionne R. A. (1989). Proglumide potentiates morphine analgesia for acute postsurgical pain. Clin. Pharmacol. Ther. 45 (6), 666–673. 10.1038/clpt.1989.88 2659236

[B146] Levine A. S. Kneip J. Grace M. Morley J. E. (1983). Effect of centrally administered neurotensin on multiple feeding paradigms. Pharmacol. Biochem. Behav. 18 (1), 19–23. 10.1016/0091-3057(83)90244-7 6131439

[B148] Levy D. (2012). Endogenous mechanisms underlying the activation and sensitization of meningeal nociceptors: the role of immuno-vascular interactions and cortical spreading depression. Curr. Pain Headache Rep. 16 (3), 270–277. 10.1007/s11916-012-0255-1 22328144

[B149] Li J.-N. Sheets P. L. (2018). The central amygdala to periaqueductal gray pathway comprises intrinsically distinct neurons differentially affected in a model of inflammatory pain. J. Physiology 596 (24), 6289–6305. 10.1113/JP276935 30281797 PMC6292805

[B150] Li A. H. Hwang H. M. Tan P. P. Wu T. Wang H. L. (2001). Neurotensin excites periaqueductal gray neurons projecting to the rostral ventromedial medulla. J. Neurophysiology 85 (4), 1479–1488. 10.1152/jn.2001.85.4.1479 11287471

[B151] Li J. Song J. Zaytseva Y. Y. Liu Y. Rychahou P. Jiang K. (2016). An obligatory role for neurotensin in high-fat-diet-induced obesity. Nature 533 (7603), 411–415. 10.1038/nature17662 27193687 PMC5484414

[B152] Li H. Wang R. Lu Y. Xu X. Ni J. (2017). Targeting G protein-coupled receptor for pain management. Brain Circ. 3 (2), 109–113. 10.4103/bc.bc_3_17 30276310 PMC6126263

[B153] Li L. Weiss H. L. Li J. Chen Z. Donato L. Evers B. M. (2020). High plasma levels of pro-NT are associated with increased colon cancer risk. Endocrine-Related Cancer 27 (11), 641–646. 10.1530/ERC-20-0310 33055301 PMC7709962

[B154] Li H. Namburi P. Olson J. M. Borio M. Lemieux M. E. Beyeler A. (2022). Neurotensin orchestrates valence assignment in the amygdala. Nature 608 (7923), 586–592. 10.1038/s41586-022-04964-y PMC9583860 35859170

[B156] Lipp J. (1991). Possible mechanisms of morphine analgesia. Clin. Neuropharmacology 14 (2), 131–147. 10.1097/00002826-199104000-00003 1849794

[B158] Lopez R. Young S. L. Cox V. C. (1991). Analgesia for formalin-induced pain by lateral hypothalamic stimulation. Brain Res. 563 (1–2), 1–6. 10.1016/0006-8993(91)91506-v 1786523

[B159] Lu D. Uldry Lavergne C. G. Choi S. Park J. Kim J. Zhao S. (2024). General anesthesia activates a central anxiolytic center in the BNST. Cell Reports 43 (11), 114909. 10.1016/j.celrep.2024.114909 PMC11645888 39460938

[B160] Ma L. Liu S. Yi M. Wan Y. (2022). Spontaneous pain as a challenge of research and management in chronic pain. Med. Review 2(3), 308–319. 10.1515/mr-2022-0007 PMC10388751 37724190

[B161] Maeno H. Yamada K. Santo-Yamada Y. Aoki K. Sun Y. J. Sato E. (2004). Comparison of mice deficient in the high- or low-affinity neurotensin receptors, Ntsr1 or Ntsr2, reveals a novel function for Ntsr2 in thermal nociception. Brain Res. 998 (1), 122–129. 10.1016/j.brainres.2003.11.039 14725975

[B162] Mai L. Liu Q. Huang F. He H. Fan W. (2021). Involvement of mast cells in the pathophysiology of pain. Front. Cell. Neurosci. 15, 665066. 10.3389/fncel.2021.665066 PMC8222580 34177465

[B163] Makoui M. H. Fekri S. Makoui R. H. Ansari N. Esmaeilzadeh A. (2025). The role of mast cells in the development and advancement of endometriosis. Am. J. Reproductive Immunol. 93 (3), e70019. 10.1111/aji.70019 40028674

[B164] Markovic T. Pedersen C. E. Massaly N. Vachez Y. M. Ruyle B. Murphy C. A. (2021). Pain induces adaptations in ventral tegmental area dopamine neurons to drive anhedonia-like behavior. Nat. Neurosci. 24 (11), 1601–1613. 10.1038/s41593-021-00924-3 PMC8556343 34663957

[B165] Martenson M. E. Cetas J. S. Heinricher M. M. (2009). A possible neural basis for stress-induced hyperalgesia. Pain 142 (3), 236–244. 10.1016/j.pain.2009.01.011 19232470 PMC2676435

[B166] Martin L. Ibrahim M. Gomez K. Yu J. Cai S. Chew L. A. (2022). Conotoxin contulakin-G engages a neurotensin receptor 2/R-type calcium channel (Cav2.3) pathway to mediate spinal antinociception. Pain 163 (9), 1751–1762. 10.1097/j.pain.0000000000002561 35050960 PMC9198109

[B167] Martin L. F. Almuslim M. Ismail K. A. Ibrahim M. M. Moutal A. Cheng K. (2025). The conotoxin Contulakin-G reverses hypersensitivity observed in rodent models of cancer-induced bone pain without inducing tolerance or motor disturbance. Pain 166 (2), 376–387. 10.1097/j.pain.0000000000003391 39297754 PMC11723812

[B168] Mason B. N. Avona A. Lackovic J. Dussor G. (2021). Dural stimulation and periorbital von frey testing in mice as a preclinical model of headache. J. of Vis. Exp. Prepr. 173, e62867. 10.3791/62867 PMC9219585 34398161

[B169] Matthes H. W. Maldonado R. Simonin F. Valverde O. Slowe S. Kitchen I. (1996). Loss of morphine-induced analgesia, reward effect and withdrawal symptoms in mice lacking the mu-opioid-receptor gene. Nature 383 (6603), 819–823. 10.1038/383819a0 8893006

[B170] Mayer D. J. Wolfle T. L. Akil H. Carder B. Liebeskind J. C. (1971). Analgesia from electrical stimulation in the brainstem of the rat. Science 174 (4016), 1351–1354. 10.1126/science.174.4016.1351 5167502

[B171] Mazella J. (2022). Deciphering mechanisms of action of sortilin/neurotensin Receptor-3 in the proliferation regulation of colorectal and other cancers. Int. J. Mol. Sci. 23 (19), 11888. 10.3390/ijms231911888 36233189 PMC9570473

[B172] Mazella J. Chabry J. Zsurger N. Vincent J. P. (1989). Purification of the neurotensin receptor from mouse brain by affinity chromatography. J. Biol. Chem. 264 (10), 5559–5563. 2538423

[B173] McCallion A. Nasirzadeh Y. Lingegowda H. Miller J. E. Khalaj K. Ahn S. (2022). Estrogen mediates inflammatory role of mast cells in endometriosis pathophysiology. Front. Immunol. 13, 961599. 10.3389/fimmu.2022.961599 36016927 PMC9396281

[B174] McClain S. P. Ma X. Johnson D. A. Johnson C. A. Layden A. E. Yung J. C. (2023). *In vivo* photopharmacology with light-activated opioid drugs. Neuron 111 (24), 3926–3940. 10.1016/j.neuron.2023.09.017 37848025 PMC11188017

[B175] McLaughlin I. Dani J. A. De Biasi M. (2017). The medial habenula and interpeduncular nucleus circuitry is critical in addiction, anxiety, and mood regulation. J. Neurochem. 142 (Suppl. 2), 130–143. 10.1111/jnc.14008 28791703 PMC6740332

[B176] Meacham K. Shepherd A. Mohapatra D. P. Haroutounian S. (2017). Neuropathic pain: central vs. peripheral mechanisms. Curr. Pain Headache Rep. 21 (6), 28. 10.1007/s11916-017-0629-5 28432601

[B177] Mechanic J. A. Sutton J. E. Berson A. E. Wu X. Kwan J. Schreiber R. (2009). Involvement of the neurotensin receptor 1 in the behavioral effects of two neurotensin agonists, NT-2 and NT69L: lack of hypothermic, antinociceptive and antipsychotic actions in receptor knockout mice. Eur. Neuropsychopharmacol. 19 (7), 466–475. 10.1016/j.euroneuro.2009.01.004 19223157

[B178] Miller L. A. Cochrane D. E. Feldberg R. S. Carraway R. E. (1998). Inhibition of neurotensin-stimulated mast cell secretion and carboxypeptidase A activity by the peptide inhibitor of carboxypeptidase A and neurotensin-receptor antagonist SR 48692. Int. Archives Allergy Immunol. 116 (2), 147–153. 10.1159/000023938 9652308

[B179] Mills A. Demoliou-Mason C. D. Barnard E. A. (1988). Purification of the neurotensin receptor from bovine brain. J. Biol. Chem. 263 (1), 13–16. 2826433

[B180] Minami M. (2009). Chapter 10 neuronal mechanisms for pain?induced aversion. Elsevier Int. Review Neurobiology, 85 135–144. 10.1016/S0074-7742(09)85010-1 19607966

[B181] Mitchell V. A. Kawahara H. Vaughan C. W. (2009). Neurotensin inhibition of GABAergic transmission *via* mGluR-induced endocannabinoid signalling in rat periaqueductal grey. J. Physiology 587 (11), 2511–2520. 10.1113/jphysiol.2008.167429 19359367 PMC2714017

[B182] Mogil J. S. (2019). The translatability of pain across species. Philosophical Trans. R. Soc. Lond. Ser. B, Biol. Sci. 374 (1785), 20190286. 10.1098/rstb.2019.0286 31544615 PMC6790385

[B183] Moretti S. Bozza S. Oikonomou V. Renga G. Casagrande A. Iannitti R. G. (2014). IL-37 inhibits inflammasome activation and disease severity in murine aspergillosis. PLoS Pathog. 10 (11), e1004462. 10.1371/journal.ppat.1004462 25375146 PMC4223056

[B184] Morgan M. M. Heinricher M. M. Fields H. L. (1992). Circuitry linking opioid-sensitive nociceptive modulatory systems in periaqueductal gray and spinal cord with rostral ventromedial medulla. Neuroscience 47 (4), 863–871. 10.1016/0306-4522(92)90036-2 1579215

[B186] Navarro V. Vincent J.-P. Mazella J. (2002). Shedding of the luminal domain of the neurotensin receptor-3/sortilin in the HT29 cell line. Biochem. Biophysical Res. Commun. 298 (5), 760–764. 10.1016/s0006-291x(02)02564-0 12419319

[B187] Navarro-Gonzalez M. C. Lerma C. López-Trejo F. I. Aranda-Cano E. Salgado-Aguayo A. Paz-Gómez D. (2025). Correlation between nocturnal heart rate variability parameters and circulating neuropeptides in patients suffering from fibromyalgia. Clin. Exp. Rheumatology 43 (6), 1035–1039. 10.55563/clinexprheumatol/rfy9hb 40242913

[B188] Nemeroff C. B. Bissette G. Prange A. J. Loosen P. T. Barlow T. S. Lipton M. A. (1977). Neurotensin: central nervous system effects of a hypothalamic peptide. Brain Res. 128 (3), 485–496. 10.1016/0006-8993(77)90173-1 406965

[B189] Neubert M. J. Kincaid W. Heinricher M. M. (2004). Nociceptive facilitating neurons in the rostral ventromedial medulla. Pain 110 (1–2), 158–165. 10.1016/j.pain.2004.03.017 15275763

[B190] Neugebauer V. Mazzitelli M. Cragg B. Ji G. Navratilova E. Porreca F. (2020). Amygdala, neuropeptides, and chronic pain-related affective behaviors. Neuropharmacology 170, 108052. 10.1016/j.neuropharm.2020.108052 32188569 PMC7214122

[B191] Neyama H. Wu Y. Nakaya Y. Kato S. Shimizu T. Tahara T. (2025). Opioidergic activation of the descending pain inhibitory system underlies placebo analgesia. Sci. Adv. 11 (3), eadp8494. 10.1126/sciadv.adp8494 39813331 PMC11734720

[B192] Ng S. K. Urquhart D. M. Fitzgerald P. B. Cicuttini F. M. Hussain S. M. Fitzgibbon B. M. (2018). The relationship between structural and functional brain changes and altered emotion and cognition in chronic low back pain brain changes: a systematic review of MRI and fMRI studies. Clin. J. Pain 34 (3), 237–261. 10.1097/AJP.0000000000000534 28719509

[B193] North R. Y. Li Y. Ray P. Rhines L. D. Tatsui C. E. Rao G. (2019). Electrophysiological and transcriptomic correlates of neuropathic pain in human dorsal root ganglion neurons. Brain A J. Neurology 142 (5), 1215–1226. 10.1093/brain/awz063 30887021 PMC6487328

[B194] Nouel D. Faure M. P. St Pierre J. A. Alonso R. Quirion R. Beaudet A. (1997). Differential binding profile and internalization process of neurotensin *via* neuronal and glial receptors. J. Neurosci. 17 (5), 1795–1803. 10.1523/JNEUROSCI.17-05-01795.1997 9030638 PMC6573365

[B195] Novakovic S. D. Tzoumaka E. McGivern J. G. Haraguchi M. Sangameswaran L. Gogas K. R. (1998). Distribution of the tetrodotoxin-resistant sodium channel PN3 in rat sensory neurons in normal and neuropathic conditions. J. Neurosci. 18 (6), 2174–2187. 10.1523/JNEUROSCI.18-06-02174.1998 9482802 PMC6792911

[B197] Ohara S. Lenz F. A. (2003). Medial lateral extent of thermal and pain sensations evoked by microstimulation in somatic sensory nuclei of human thalamus. J. Neurophysiology 90 (4), 2367–2377. 10.1152/jn.00450.2003 12840083

[B198] Osbahr A. J. Nemeroff C. B. Luttinger D. Mason G. A. Prange A. J. (1981). Neurotensin-induced antinociception in mice: antagonism by thyrotropin-releasing hormone. J. Pharmacol. Exp. Ther. 217 (3), 645–651. 6112261

[B199] Ossipov M. H. Dussor G. O. Porreca F. (2010). Central modulation of pain. J. Clin. Investigation 120 (11), 3779–3787. 10.1172/JCI43766 21041960 PMC2964993

[B200] Ossipov M. H. Morimura K. Porreca F. (2014). Descending pain modulation and chronification of pain. Curr. Opinion Supportive Palliative Care 8 (2), 143–151. 10.1097/SPC.0000000000000055 24752199 PMC4301419

[B201] Paul A. K. Smith C. M. Rahmatullah M. Nissapatorn V. Wilairatana P. Spetea M. (2021). Opioid analgesia and opioid-induced adverse effects: a review. Pharm. Basel, Switz. 14 (11), 1091. 10.3390/ph14111091 PMC8620360 34832873

[B202] Peng B. Jiao Y. Zhang Y. Li S. Chen S. Xu S. (2023). Bulbospinal nociceptive ON and OFF cells related neural circuits and transmitters. Front. Pharmacology 14, 1159753. 10.3389/fphar.2023.1159753 PMC10157642 37153792

[B203] Petkova-Kirova P. Rakovska A. Della Corte L. Zaekova G. Radomirov R. Mayer A. (2008). Neurotensin modulation of acetylcholine, GABA, and aspartate release from rat prefrontal cortex studied *in vivo* with microdialysis. Brain Res. Bull. 77 (2–3), 129–135. 10.1016/j.brainresbull.2008.04.003 18721670

[B204] Pettibone D. J. Hess J. F. Hey P. J. Jacobson M. A. Leviten M. Lis E. V. (2002). The effects of deleting the mouse neurotensin receptor NTR1 on central and peripheral responses to neurotensin. J. Pharmacol. Exp. Ther. 300 (1), 305–313. 10.1124/jpet.300.1.305 11752130

[B205] Piliponsky A. M. Chen C. C. Nishimura T. Metz M. Rios E. J. Dobner P. R. (2008). Neurotensin increases mortality and mast cells reduce neurotensin levels in a mouse model of sepsis. Nat. Med. 14 (4), 392–398. 10.1038/nm1738 18376408 PMC2873870

[B206] Pinho-Ribeiro F. A. Verri W. A. Chiu I. M. (2017). Nociceptor sensory neuron-immune interactions in pain and inflammation. Trends Immunol. 38 (1), 5–19. 10.1016/j.it.2016.10.001 27793571 PMC5205568

[B208] Potrebic S. B. Mason P. Fields H. L. (1995). The density and distribution of serotonergic appositions onto identified neurons in the rat rostral ventromedial medulla. J. Neurosci. 15 (5 Pt 1), 3273–3283. 10.1523/JNEUROSCI.15-05-03273.1995 7751910 PMC6578240

[B209] Pulskamp T. G. Johnson L. M. Berlau D. J. (2024). Novel non-opioid analgesics in pain management. Pain Management 14 (12), 641–651. 10.1080/17581869.2024.2442292 39692452 PMC11702995

[B210] Qing-Ping W. Nakai Y. (1994). The dorsal raphe: an important nucleus in pain modulation. Brain Res. Bull. 34 (6), 575–585. 10.1016/0361-9230(94)90143-0 7922601

[B211] Qiu D. Zhou Z. B. Li X. Y. Hashimoto K. Yang J. J. Wang X. M. (2026). Chronic pain and risk of cognitive impairment: a meta-analysis of longitudinal cohort studies. Transl. Psychiatry 16 (1), 133. 10.1038/s41398-026-03924-8 41792103 PMC12976088

[B212] Raja S. N. Carr D. B. Cohen M. Finnerup N. B. Flor H. Gibson S. (2020). The revised international association for the study of pain definition of pain: concepts, challenges, and compromises. Pain 161 (9), 1976–1982. 10.1097/j.pain.0000000000001939 32694387 PMC7680716

[B213] Rakovska A. Giovannini M. G. Della Corte L. Kalfin R. Bianchi L. Pepeu G. (1998). Neurotensin modulation of acetylcholine and GABA release from the rat hippocampus: an *in vivo* microdialysis study. Neurochem. Int. 33 (4), 335–340. 10.1016/s0197-0186(98)00036-9 9840224

[B214] Reinecke M. Forssmann W. G. Thiekötter G. Triepel J. (1983). Localization of neurotensin-immunoreactivity in the spinal cord and peripheral nervous system of the Guinea pig. Neurosci. Lett. 37 (1), 37–42. 10.1016/0304-3940(83)90501-3 6877658

[B215] Remaury A. Vita N. Gendreau S. Jung M. Arnone M. Poncelet M. (2002). Targeted inactivation of the neurotensin type 1 receptor reveals its role in body temperature control and feeding behavior but not in analgesia. Brain Res. 953 (1–2), 63–72. 10.1016/s0006-8993(02)03271-7 12384239

[B216] Reynolds D. V. (1969). Surgery in the rat during electrical analgesia induced by focal brain stimulation. Science 164 (3878), 444–445. 10.1126/science.164.3878.444 4887743

[B217] Rikard S. M. Strahan A. E. Schmit K. M. Guy G. P. Jr. (2023). Chronic pain among adults - united States, 2019-2021. MMWR. Morb. Mortal. Wkly. Rep. 72 (15), 379–385. 10.15585/mmwr.mm7215a1 37053114 PMC10121254

[B219] Roussy G. Dansereau M. A. Doré-Savard L. Belleville K. Beaudet N. Richelson E. (2008). Spinal NTS1 receptors regulate nociceptive signaling in a rat formalin tonic pain model. J. Neurochem. 105 (4), 1100–1114. 10.1111/j.1471-4159.2007.05205.x 18182046

[B220] Roussy G. Dansereau M. A. Baudisson S. Ezzoubaa F. Belleville K. Beaudet N. (2009). Evidence for a role of NTS2 receptors in the modulation of tonic pain sensitivity. Mol. Pain 5, 38. 10.1186/1744-8069-5-38 19580660 PMC2714839

[B221] Roussy G. Beaudry H. Lafrance M. Belleville K. Beaudet N. Wada K. (2010). Altered morphine-induced analgesia in neurotensin type 1 receptor null mice. Neuroscience 170 (4), 1286–1294. 10.1016/j.neuroscience.2010.08.016 20727387 PMC3848973

[B222] Russell F. A. King R. Smillie S. J. Kodji X. Brain S. D. (2014). Calcitonin gene-related peptide: physiology and pathophysiology. Physiol. Rev. 94 (4), 1099–1142. 10.1152/physrev.00034.2013 25287861 PMC4187032

[B223] Sadler K. E. Mogil J. S. Stucky C. L. (2022). Innovations and advances in modelling and measuring pain in animals. Nat. Rev. Neurosci. 23 (2), 70–85. 10.1038/s41583-021-00536-7 34837072 PMC9098196

[B224] Salahi M. Parsa S. Nourmohammadi D. Razmkhah Z. Salimi O. Rahmani M. (2022). Immunologic aspects of migraine: a review of literature. Front. Neurology 13, 944791. 10.3389/fneur.2022.944791 PMC9554313 36247795

[B225] Salio C. Lossi L. Ferrini F. Merighi A. (2006). Neuropeptides as synaptic transmitters. Cell Tissue Res. 326 (2), 583–598. 10.1007/s00441-006-0268-3 16847638

[B226] Samineni V. K. Grajales-Reyes J. G. Copits B. A. O'Brien D. E. Trigg S. L. Gomez A. M. (2017). Divergent modulation of nociception by glutamatergic and gabaergic neuronal subpopulations in the periaqueductal gray. eNeuro 4 (2). 10.1523/ENEURO.0129-16.2017 28374016 PMC5370278

[B227] Samsam M. Coveñas Rodríguez R. Yajeya Pérez J. Ahangari R. Narváez Bueno J. Á. Montes Gonzalo M. C. (2002). Implicación de los neuropéptidos metioninaencefalina, neurotensina y somatostatina del núcleo caudal del trigémino en la migraña experimental. Rev. Neurol. 34 (08), 724. 10.33588/rn.3408.2001451 12080490

[B228] Sang C. N. Barnabe K. J. Kern S. E. (2016). Phase IA clinical trial evaluating the tolerability, pharmacokinetics, and analgesic efficacy of an intrathecally administered neurotensin A analogue in central neuropathic pain following spinal cord injury. Clin. Pharmacology Drug Development 5 (4), 250–258. 10.1002/cpdd.253 27310326

[B229] Sarret P. Perron A. Stroh T. Beaudet A. (2003). Immunohistochemical distribution of NTS2 neurotensin receptors in the rat central nervous system. J. Comp. Neurology 461 (4), 520–538. 10.1002/cne.10718 12746866

[B230] Sarret P. Esdaile M. J. Perron A. Martinez J. Stroh T. Beaudet A. (2005). Potent spinal analgesia elicited through stimulation of NTS2 neurotensin receptors. J. Neurosci. 25 (36), 8188–8196. 10.1523/JNEUROSCI.0810-05.2005 16148226 PMC6725526

[B231] Sato D. Narita M. Hamada Y. Mori T. Tanaka K. Tamura H. (2022). Relief of neuropathic pain by cell-specific manipulation of nucleus accumbens dopamine D1-and D2-receptor-expressing neurons. Mol. Brain 15 (1), 10. 10.1186/s13041-021-00896-2 34991655 PMC8740378

[B232] Schmidt B. L. De Logu F. Nassini R. Geppetti P. Bunnett N. W. (2025). Pain signaling by gpcrs and rtks. Trends Pharmacol. Sci. 46 (4), 372–385. 10.1016/j.tips.2025.02.002 40057436 PMC11972155

[B233] Schroeder L. E. Leinninger G. M. (2018). Role of central neurotensin in regulating feeding: implications for the development and treatment of body weight disorders. Biochimica Biophysica Acta. Mol. Basis Dis. 1864 (3), 900–916. 10.1016/j.bbadis.2017.12.036 29288794 PMC5803395

[B234] Schroeder L. E. Furdock R. Quiles C. R. Kurt G. Perez-Bonilla P. Garcia A. (2019). Mapping the populations of neurotensin neurons in the Male mouse brain. Neuropeptides 76, 101930. 10.1016/j.npep.2019.05.001 31079844 PMC7721284

[B235] Schulz S. Röcken C. Ebert M. P. A. Schulz S. (2006). Immunocytochemical identification of low-affinity NTS2 neurotensin receptors in parietal cells of human gastric mucosa. J. Endocrinol. 191 (1), 121–128. 10.1677/joe.1.06903 17065395

[B236] Sherafat Y. Bautista M. Fowler J. P. Chen E. Ahmed A. Fowler C. D. (2020). The interpeduncular-ventral hippocampus pathway mediates active stress coping and natural reward. eNeuro 7 (6). 10.1523/ENEURO.0191-20.2020 33139320 PMC7688303

[B237] Shi T.-J. S. Xiang Q. Zhang M. D. Barde S. Kai-Larsen Y. Fried K. (2014). Somatostatin and its 2A receptor in dorsal root ganglia and dorsal horn of mouse and human: expression, trafficking and possible role in pain. Mol. Pain 10, 12. 10.1186/1744-8069-10-12 24521084 PMC3943448

[B238] Shipley M. T. McLean J. H. Behbehani M. M. (1987). Heterogeneous distribution of neurotensin-like immunoreactive neurons and fibers in the midbrain periaqueductal gray of the rat. J. Neurosci. 7 (7), 2025–2034. 10.1523/JNEUROSCI.07-07-02025.1987 3302124 PMC6568941

[B239] Shiva N. Kawase H. Einsiedel J. Kwon J. Kumar S. Shao J. (2026). Neurotensin receptor 2 agonism attenuates adverse cardiac remodeling in preclinical models. Sci. Transl. Med. 18 (856), eaea9877. 10.1126/scitranslmed.aea9877 42384771

[B240] Shrestha M. Singh R. Moreden J. Hayes J. E. (1996). Ketorolac vs chlorpromazine in the treatment of acute migraine without aura. A prospective, randomized, double-blind trial. Archives Intern. Med. 156 (15), 1725–1728. 8694672

[B241] Slosky L. M. Bai Y. Toth K. Ray C. Rochelle L. K. Badea A. (2020). β-Arrestin-Biased allosteric modulator of NTSR1 selectively attenuates addictive behaviors. Cell 181 (6), 1364–1379.e14. 10.1016/j.cell.2020.04.053 32470395 PMC7466280

[B242] Smith K. E. Boules M. Williams K. Richelson E. (2012). NTS1 and NTS2 mediate analgesia following neurotensin analog treatment in a mouse model for visceral pain. Behav. Brain Res. 232 (1), 93–97. 10.1016/j.bbr.2012.03.044 22504145

[B243] Song L. Ge T. Cui M. Zhang M. Wu M. Li Q. (2026). An NTS-Expressing inhibitory circuit from interpeduncular nucleus to dorsal raphe nucleus controls pain and comorbid depression in male mice. Cell Reports 45 (1), 116719. 10.1016/j.celrep.2025.116719 41411126

[B244] St-Gelais F. Jomphe C. Trudeau L.-E. (2006). The role of neurotensin in central nervous system pathophysiology: what is the evidence? J. Psychiatry and Neurosci. 31 (4), 229–245. 16862241 PMC1488904

[B245] Stanzione P. Zieglgänsberger W. (1983). Action of neurotensin on spinal cord neurons in the rat. Brain Res. 268 (1), 111–118. 10.1016/0006-8993(83)90395-5 6860954

[B246] Steiner T. J. Stovner L. J. Vos T. (2016). GBD 2015: migraine is the third cause of disability in under 50s. J. Headache Pain 17 (1), 104. 10.1186/s10194-016-0699-5 27844455 PMC5108738

[B247] Sullivan K. A. Hayes J. M. Wiggin T. D. Backus C. Su Oh S. Lentz S. I. (2007). Mouse models of diabetic neuropathy. Neurobiol. Dis. 28 (3), 276–285. 10.1016/j.nbd.2007.07.022 17804249 PMC3730836

[B248] Tabarean I. V. (2020). Neurotensin induces hypothermia by activating both neuronal neurotensin receptor 1 and astrocytic neurotensin receptor 2 in the median preoptic nucleus. Neuropharmacology 171, 108069. 10.1016/j.neuropharm.2020.108069 32275927 PMC7274271

[B249] Takahashi D. Asaoka Y. Kimura K. Hara R. Arakaki S. Sakasai K. (2019). Tonic suppression of the mesolimbic dopaminergic system by enhanced corticotropin-releasing factor signaling within the bed nucleus of the stria terminalis in chronic pain model rats. J. Neurosci. 39 (42), 8376–8385. 10.1523/JNEUROSCI.3047-18.2019 31451580 PMC6794933

[B250] Tanaka K. Masu M. Nakanishi S. (1990). Structure and functional expression of the cloned rat neurotensin receptor. Neuron 4 (6), 847–854. 10.1016/0896-6273(90)90137-5 1694443

[B251] Tao Z.-Y. Wang P. X. Wei S. Q. Traub R. J. Li J. F. Cao D. Y. (2019). The role of descending pain modulation in chronic primary pain: potential application of drugs targeting serotonergic system. Neural Plast. 2019, 1389296. 10.1155/2019/1389296 31933624 PMC6942873

[B252] Tappe-Theodor A. King T. Morgan M. M. (2019). Pros and cons of clinically relevant methods to assess pain in rodents. Neurosci. Biobehav. Rev. 100, 335–343. 10.1016/j.neubiorev.2019.03.009 30885811 PMC6528820

[B253] Terrier L.-M. Hadjikhani N. Destrieux C. (2022). The trigeminal pathways. J. Neurology 269 (7), 3443–3460. 10.1007/s00415-022-11002-4 35249132

[B254] Tétreault P. Beaudet N. Perron A. Belleville K. René A. Cavelier F. (2013). Spinal NTS2 receptor activation reverses signs of neuropathic pain. FASEB J. 27 (9), 3741–3752. 10.1096/fj.12-225540 23756650

[B255] Theoharides T. C. Zhang B. Kempuraj D. Tagen M. Vasiadi M. Angelidou A. (2010). IL-33 augments substance P-induced VEGF secretion from human mast cells and is increased in psoriatic skin. Proc. Natl. Acad. Sci. U. S. A. 107 (9), 4448–4453. 10.1073/pnas.1000803107 20160089 PMC2840132

[B256] Theoharides T. C. Alysandratos K. D. Angelidou A. Delivanis D. A. Sismanopoulos N. Zhang B. (2012). Mast cells and inflammation. Biochimica Biophysica Acta 1822 (1), 21–33. 10.1016/j.bbadis.2010.12.014 21185371 PMC3318920

[B257] Theoharides T. C. Tsilioni I. Arbetman L. Panagiotidou S. Stewart J. M. Gleason R. M. (2015). Fibromyalgia syndrome in need of effective treatments. J. Pharmacol. Exp. Ther. 355 (2), 255–263. 10.1124/jpet.115.227298 26306765 PMC4613957

[B258] Theoharides T. C. Tsilioni I. Bawazeer M. (2019). Mast cells, neuroinflammation and pain in fibromyalgia syndrome. Front. Cell. Neurosci. 13, 353. 10.3389/fncel.2019.00353 31427928 PMC6687840

[B259] Thompson K. D. Leinninger G. M. (2025). Influence of the neurotensin signaling system on feeding and satiety. Neuropharmacology 275, 110496. 10.1016/j.neuropharm.2025.110496 40324649 PMC12109131

[B260] Thompson J. M. Neugebauer V. (2017). “Amygdala plasticity and pain.,” Pain Res. Manag., 2017. 8296501. 10.1155/2017/8296501 29302197 PMC5742506

[B261] Thor K. Rosell S. (1986). Neurotensin increases colonic motility. Gastroenterology 90 (1), 27–31. 10.1016/0016-5085(86)90070-3 3940251

[B262] Torruella-Suárez M. L. McElligott Z. A. (2020). Neurotensin in reward processes. Neuropharmacology 167, 108005. 10.1016/j.neuropharm.2020.108005 32057800 PMC7238864

[B263] Treede R. D. Rolke R. Andrews K. Magerl W. (2002). Pain elicited by blunt pressure: neurobiological basis and clinical relevance. Pain 98 (3), 235–240. 10.1016/S0304-3959(02)00203-8 12127024

[B264] Tsai H. Y. Maeda S. Iwatsubo K. Inoki R. (1984). Effect of neuroactive peptides on labeled 5-hydroxytryptamine release from rat spinal slices *in vitro* . Jpn. Journal Pharmacology 35 (4), 403–406. 10.1254/jjp.35.403 6209442

[B265] Tschumi C. W. Beckstead M. J. (2019). Diverse actions of the modulatory peptide neurotensin on central synaptic transmission. Eur. J. Neurosci. 49 (6), 784–793. 10.1111/ejn.13858 29405480 PMC6078827

[B266] Tsilioni I. Russell I. J. Stewart J. M. Gleason R. M. Theoharides T. C. (2016). Neuropeptides CRH, SP, HK-1, and inflammatory cytokines IL-6 and TNF are increased in serum of patients with fibromyalgia syndrome, implicating mast cells. J. Pharmacol. Exp. Ther. 356 (3), 664–672. 10.1124/jpet.115.230060 26763911 PMC4767394

[B267] Uhl G. R. Bennett J. P. Snyder S. H. (1977a). Neurotensin, a central nervous system peptide: apparent receptor binding in brain membranes. Brain Res. 130 (2), 299–313. 10.1016/0006-8993(77)90277-3 884526

[B268] Uhl G. R. Kuhar M. J. Snyder S. H. (1977b). Neurotensin: immunohistochemical localization in rat central nervous system. Proc. Natl. Acad. Sci. U. S. A. 74 (9), 4059–4063. 10.1073/pnas.74.9.4059 333458 PMC431844

[B269] Uhl G. R. Goodman R. R. Snyder S. H. (1979). Neurotensin-containing cell bodies, fibers and nerve terminals in the brain stem of the rat: immunohistochemical mapping. Brain Res. 167 (1), 77–91. 10.1016/0006-8993(79)90264-6 378326

[B270] Uniyal A. Tiwari V. Tsukamoto T. Dong X. Guan Y. Raja S. N. (2023). Targeting sensory neuron GPCRs for peripheral neuropathic pain. Trends Pharmacol. Sci. 44 (12), 1009–1027. 10.1016/j.tips.2023.10.003 37977131 PMC10657387

[B271] Urban M. O. Smith D. J. (1993). Role of neurotensin in the nucleus raphe magnus in opioid-induced antinociception from the periaqueductal gray. J. Pharmacol. Exp. Ther. 265 (2), 580–586. 8496808

[B272] van den Pol A. N. (2012). Neuropeptide transmission in brain circuits. Neuron 76 (1), 98–115. 10.1016/j.neuron.2012.09.014 23040809 PMC3918222

[B273] Vaughan C. W. Ingram S. L. Connor M. A. Christie M. J. (1997). How opioids inhibit GABA-Mediated neurotransmission. Nature 390 (6660), 611–614. 10.1038/37610 9403690

[B274] Vincent J. P. Mazella J. Kitabgi P. (1999). Neurotensin and neurotensin receptors. Trends Pharmacol. Sci. 20 (7), 302–309. 10.1016/s0165-6147(99)01357-7 10390649

[B275] Vita N. Laurent P. Lefort S. Chalon P. Dumont X. Kaghad M. (1993). Cloning and expression of a complementary DNA encoding a high affinity human neurotensin receptor. FEBS Lett. 317 (1–2), 139–142. 10.1016/0014-5793(93)81509-x 8381365

[B276] Vivancos M. Fanelli R. Besserer-Offroy É. Beaulieu S. Chartier M. Resua-Rojas M. (2021). Metabolically stable neurotensin analogs exert potent and long-acting analgesia without hypothermia. Behav. Brain Res. 405, 113189. 10.1016/j.bbr.2021.113189 33607165

[B277] Volkow N. D. McLellan A. T. (2016). Opioid abuse in chronic Pain--Misconceptions and mitigation strategies. N. Engl. J. Med. 374 (13), 1253–1263. 10.1056/NEJMra1507771 27028915

[B278] Von Korff M. R. (2013). Long-term use of opioids for complex chronic pain. Best Pract. and Res. Clin. Rheumatology 27 (5), 663–672. 10.1016/j.berh.2013.09.011 24315147 PMC4036624

[B279] Walters E. T. Crook R. J. Neely G. G. Price T. J. Smith E. S. J. (2023). Persistent nociceptor hyperactivity as a painful evolutionary adaptation. Trends Neurosci. 46 (3), 211–227. 10.1016/j.tins.2022.12.007 36610893 PMC9974896

[B280] Wang J. Zhang H. Feng Y. P. Meng H. Wu L. P. Wang W. (2014). Morphological evidence for a neurotensinergic periaqueductal gray-rostral ventromedial medulla-spinal dorsal horn descending pathway in rat. Front. Neuroanat. 8, 112. 10.3389/fnana.2014.00112 25346662 PMC4191475

[B281] Wang S. Veinot J. Goyal A. Khatibi A. Lazar S. W. Hashmi J. A. (2022). Distinct networks of periaqueductal gray columns in pain and threat processing. Neuroimage 250, 118936. 10.1016/j.neuroimage.2022.118936 35093518

[B282] Wang D. Pan X. Zhou Y. Wu Z. Ren K. Liu H. (2023). Lateral septum-lateral hypothalamus circuit dysfunction in comorbid pain and anxiety. Mol. Psychiatry 28 (3), 1090–1100. 10.1038/s41380-022-01922-y 36642737 PMC10005966

[B283] Wang J. Mao X. Zhu L. Zhang X. (2025). Unravelling the intricate link: mast cells and estrogen-induced pain sensitization in endometriosis. Int. J. Biol. Sci. 21 (13), 5891–5904. 10.7150/ijbs.116635 41079937 PMC12509920

[B284] Wang S. Guo Y. Wei B. Lu R. Tan Z. Wei C. (2025). Dopaminergic neurons in the ventral periaqueductal gray projecting to the dorsal lateral septum regulate comorbid pain and anxiety. Brain Res. Bull. 228, 111409. 10.1016/j.brainresbull.2025.111409 40449627

[B285] Watters J. J. Dorsa D. M. (1998). Transcriptional effects of estrogen on neuronal neurotensin gene expression involve cAMP/protein kinase A-dependent signaling mechanisms. J. Neurosci. 18 (17), 6672–6680. 10.1523/JNEUROSCI.18-17-06672.1998 9712639 PMC6792960

[B286] Weissman-Fogel I. Sprecher E. Granovsky Y. Yarnitsky D. (2003). Repeated noxious stimulation of the skin enhances cutaneous pain perception of migraine patients in-between attacks: clinical evidence for continuous sub-threshold increase in membrane excitability of central trigeminovascular neurons. Pain 104 (3), 693–700. 10.1016/S0304-3959(03)00159-3 12927642

[B287] Williams F. G. Beitz A. J. (1993). Chronic pain increases brainstem proneurotensin/neuromedin-N mRNA expression: a hybridization-histochemical and immunohistochemical study using three different rat models for chronic nociception. Brain Res. 611 (1), 87–102. 10.1016/s0006-8993(93)90001-4 8518953

[B288] Williams F. G. Mullet M. A. Beitz A. J. (1995). Basal release of Met-enkephalin and neurotensin in the ventrolateral periaqueductal gray matter of the rat: a microdialysis study of antinociceptive circuits. Brain Res. 690 (2), 207–216. 10.1016/0006-8993(95)00554-4 8535838

[B289] Willis W. D. Westlund K. N. (1997). Neuroanatomy of the pain system and of the pathways that modulate pain. J. Clin. Neurophysiology 14 (1), 2–31. 10.1097/00004691-199701000-00002 9013357 PMC7859971

[B290] Woodworth H. L. Batchelor H. M. Beekly B. G. Bugescu R. Brown J. A. Kurt G. (2017). Neurotensin Receptor-1 identifies a subset of ventral tegmental dopamine neurons that coordinates energy balance. Cell Reports 20 (8), 1881–1892. 10.1016/j.celrep.2017.08.001 28834751 PMC5584591

[B291] Woodworth H. L. Brown J. A. Batchelor H. M. Bugescu R. Leinninger G. M. (2018). Determination of neurotensin projections to the ventral tegmental area in mice. Neuropeptides 68, 57–74. 10.1016/j.npep.2018.02.003 29478718 PMC5906039

[B292] Woodworth H. L. Perez-Bonilla P. A. Beekly B. G. Lewis T. J. Leinninger G. M. (2018). Identification of neurotensin receptor expressing cells in the ventral tegmental area across the lifespan. eNeuro 5 (1). 10.1523/ENEURO.0191-17.2018 29464190 PMC5815659

[B294] Xie K.-Y. Wang Q. Cao D. J. Liu J. Xie X. F. (2019). Spinal astrocytic FGFR3 activation leads to mechanical hypersensitivity by increased TNF-α in spared nerve injury. Int. Journal Clinical Experimental Pathology 12 (8), 2898–2908. 31934126 PMC6949693

[B295] Xie K. Cheng X. Zhu T. Zhang D. (2023). Single-cell transcriptomic profiling of dorsal root ganglion: an overview. Front. Neuroanat. 17, 1162049. 10.3389/fnana.2023.1162049 37405309 PMC10315536

[B296] Xu N. Tang X. H. Pan W. Xie Z. M. Zhang G. F. Ji M. H. (2017). Spared nerve injury increases the expression of microglia M1 markers in the prefrontal cortex of rats and provokes depression-like behaviors. Front. Neurosci. 11, 209. 10.3389/fnins.2017.00209 28458629 PMC5394168

[B297] Xu C. Sun Y. Cai X. You T. Zhao H. Li Y. (2018). Medial habenula-interpeduncular nucleus circuit contributes to anhedonia-like behavior in a rat model of depression. Front. Behav. Neurosci. 12, 238. 10.3389/fnbeh.2018.00238 30356828 PMC6189744

[B298] Xue T.-F. Ding X. Ji J. Yan H. Huang J. Y. Guo X. D. (2017). PD149163 induces hypothermia to protect against brain injury in acute cerebral ischemic rats. J. Pharmacol. Sci. 135 (3), 105–113. 10.1016/j.jphs.2017.10.004 29113791

[B299] Yamauchi N. Sato K. Sato K. Murakawa S. Hamasaki Y. Nomura H. (2022). Chronic pain-induced neuronal plasticity in the bed nucleus of the stria terminalis causes maladaptive anxiety. Sci. Adv. 8 (17), eabj5586. 10.1126/sciadv.abj5586 35476439 PMC9045713

[B300] Yen C.-T. Lu P.-L. (2013). Thalamus and pain. Acta Anaesthesiol. Taiwanica 51 (2), 73–80. 10.1016/j.aat.2013.06.011 23968658

[B301] Yen C.-T. Shaw F.-Z. (2003). Reticular thalamic responses to nociceptive inputs in anesthetized rats. Brain Res. 968 (2), 179–191. 10.1016/s0006-8993(03)02235-2 12663087

[B302] Yin M. Kim Y. O. Choi J. I. Jeong S. Yang S. H. Bae H. B. (2020). Antinociceptive role of neurotensin receptor 1 in rats with chemotherapy-induced peripheral neuropathy. Korean Journal Pain 33 (4), 318–325. 10.3344/kjp.2020.33.4.318 32989196 PMC7532295

[B303] Yong R. J. Mullins P. M. Bhattacharyya N. (2022). Prevalence of chronic pain among adults in the United States. Pain 163 (2), e328–e332. 10.1097/j.pain.0000000000002291 33990113

[B304] Zahm D. S. Grosu S. Williams E. A. Qin S. Bérod A. (2001). Neurons of origin of the neurotensinergic plexus enmeshing the ventral tegmental area in rat: retrograde labeling and *in situ* hybridization combined. Neuroscience 104 (3), 841–851. 10.1016/s0306-4522(01)00118-x 11440814

[B305] Zhang M.-M. Feng Y. P. Qiu X. T. Chen T. Bai Y. Feng J. M. (2021). Neurotensin attenuates nociception by facilitating inhibitory synaptic transmission in the mouse spinal cord. Front. Neural Circuits 15, 775215. 10.3389/fncir.2021.775215 35002634 PMC8740200

[B306] Zhang H. Li L. Zhang X. Ru G. Zang W. (2024). Role of the dorsal raphe nucleus in pain processing. Brain Sciences 14 (10), 982. 10.3390/brainsci14100982 39451996 PMC11506261

[B307] Zhang L. Ji M. Sun Y. Wang Q. Jin M. Wang S. (2025). VTA dopaminergic neurons involved in chronic spared nerve injury pain-induced depressive-like behavior. Brain Res. Bull. 222, 111261. 10.1016/j.brainresbull.2025.111261 39956400

